# Contraceptive service provider imposed restrictions to contraceptive access in urban Nigeria

**DOI:** 10.1186/s12913-017-2233-0

**Published:** 2017-04-12

**Authors:** Hilary M. Schwandt, Ilene S. Speizer, Meghan Corroon

**Affiliations:** 1 Johns Hopkins University Center for Communication Programs, Baltimore, MD USA; 2 Fairhaven College, Western Washington University, Bellingham, WA USA; 3 Carolina Population Center at The University of North Carolina at Chapel Hill, Chapel Hill, NC USA; 4 Department of Maternal and Child Health, Gillings School of Global Public Health, The University of North Carolina at Chapel Hill, Chapel Hill, NC USA

**Keywords:** Nigeria, Provider Bias, Contraception, Family Planning, Training, Urban

## Abstract

**Background:**

Health service providers can restrict access to contraceptives through their own imposed biases about method appropriateness. In this study, provider biases toward contraceptive service provision among urban Nigerian providers was assessed.

**Methods:**

Health providers working in health facilities, as well as pharmacists and patent medical vendors (PMV), in Abuja, Benin City, Ibadan, Ilorin, Kaduna, and Zaria, were surveyed in 2011 concerning their self-reported biases in service provision based on age, parity, and marital status.

**Results:**

Minimum age bias was the most common bias while minimum parity was the least common bias reported by providers. Condoms were consistently provided with the least amount of bias, followed by provision of emergency contraception (EC), pills, injectables, and IUDs. Experience of in-service training for health facility providers was associated with decreased prevalence of marital status bias for the pill, injectable, and IUD; however, training experience did not, or had the opposite effect on, pharmacists and PMV operator’s reports of service provision bias.

**Conclusions:**

Provider imposed eligibility barriers in urban study sites in Nigeria were pervasive - the most prevalent restriction across method and provider type was minimum age. Given the large and growing adolescent population - interventions aimed at increasing supportive provision of contraceptives to youth in this context are urgently needed. The results show that the effect of in-service training on provider biases was limited. Future efforts to address provider biases in contraceptive service provision, among all provider types, must find creative ways to address this critical barrier to increased contraceptive use.

**Electronic supplementary material:**

The online version of this article (doi:10.1186/s12913-017-2233-0) contains supplementary material, which is available to authorized users.

## Background

Nigeria, with a population of 187 million in 2016, is the largest country in Africa and ranks seventh in the world [1]. Nationally, Nigeria has exhibited a low (10%) and stagnating modern contraceptive prevalence rate for more than a decade but recent trends in urban areas of the country show promising increases in modern contraceptive use from 17% in 2008 [2] to 27% in 2013 [3]. Contraceptive method use in 2011 in six urban cities (Abuja, Benin City, Ibadan, Ilorin, Kaduna, and Zaria) was dominated by male condoms, followed by injectables, pills, IUDs, and emergency contraception (EC). The least commonly used methods were implants and sterilization. Most users obtained these methods from Patent Medical Vendors (PMV), followed by public sector health facilities, and pharmacies. Private sector health facilities served the fewest users in all cities [4].

Women who desire to delay or limit births often face barriers to contraceptive method use. Barriers to contraceptive method use occur on both the demand side - through a lack of awareness or education and fear of side effects - as well as on the supply side -* through contraceptive method stockouts, geographical distance, or through individual health care provider biases of who should or should not obtain family planning services.

Contraceptive service providers can increase the odds of continued contraceptive use [5] or contribute to barriers to uptake [6]. One way providers contribute to barriers to contraceptive use is through restricting access to methods based on their own personal biases about who should or shouldn’t use certain contraceptive methods [7]. Barriers to contraceptive access imposed by providers on clients with unfounded medical justifications are called “medical barriers” [8, 9].

Provider imposed medical eligibility barriers have been shown to affect access to and use of contraception in a variety of settings [10–18]. These barriers are the result of a provider deciding which contraceptive method to offer on the basis of his/her own cultural and social norms or on the basis of his/her observations about a client’s personal characteristics, such as: age, parity, and marital status.

Training of family planning providers, whether pre-service or in-service training, is one way to educate providers on how to avoid biasing service provision based on personal morals. Research on contraceptive access have included calls to increase the coverage and quality of provider training [19] - and to provide focused training in particular areas, such as provision of services to adolescents [20]. Client use of FP services in Pakistan has been associated with FP training experience of providers [21]. Prior research from Nigeria has indicated that family planning (FP) training among providers offering FP is actually rare [11].

The aim of this study was to examine the prevalence of contraceptive provision bias, by method, provider type, and training experience, among family planning providers in six Nigerian cities. This is the first study to examine provider bias toward contraceptive provision in an urban Nigerian context.

## Methods

This study relies on three reproductive health service provider surveys collected from January 2011- July 2011 in the following six Nigerian cities: Abuja, Benin City, Ibadan, Ilorin, Kaduna, and Zaria. In all public and private health facilities included, a provider survey was undertaken with doctors, nurse/midwives, and community health extension workers (CHEW). CHEWs are health workers with limited, and specific training on basic family planning services. All contraceptive methods are available at most health facilities. A separate provider survey was conducted among pharmacists. Pharmacists can provide condoms, EC, pills, and injectables. The third provider survey was with patent medical vendors (PMV) - who can only provide over the counter medications: condoms, emergency contraception (EC), and pill refills.

### Sampling

Information was collected from a census of public health facilities offering reproductive health services. A sample of private facilities was also included based upon data from a women’s individual survey conducted just prior to sampling for this study, which included specific questions on facilities visited for family planning services [4]. The overall facility sample includes service delivery points (SDPs) managed by government, private providers, nongovernmental organizations, and faith-based organizations. At each SDP, data were collected from a random sample of up to four individual health care providers who were permanently employed, medically qualified, and provided at least one clinical reproductive health service (family planning, MNCH, or HIV/AIDS/STI services). When there were more than four eligible providers in a facility on the day of the interview, a random number approach was used to select four to be approached for interview. A sample of 400 health facilities and 1,479 providers across the six cities was selected for the survey. No weighting was applied in the analysis.

The survey design called for a sample of 100 pharmacies and PMVs in each city. To obtain these samples, first lists of registered establishments of pharmacies and PMVs located within the six cities were obtained. The lists were cross-checked and compiled into a single master list for ground verification. Next, the facilities listed were physically verified in each city and, when relevant, new pharmacies or PMVs were added to the master list. After physical verification, a simple questionnaire was administered to collect the following information: name, type of facility, ownership, community, address, local government area (LGA) where the facility was located, and whether family planning was offered at the facility. Only listed pharmacies and PMVs indicating provision of family planning counseling or methods were eligible for audit. Among the listed pharmacies in Abuja and Kaduna, 100 were randomly selected for inclusion. All pharmacies in Benin City, Ibadan, Ilorin, and Zaria were included as slightly more or less than 100 eligible outlets were listed in each city. The same protocol was followed for PMVs. In all cities except for Abuja, a large number of eligible PMVs were listed and thus, a random sample of 100 were selected. Abuja had less than 100 PMVs, so all listed outlets were included in the survey sample. The final total audited sample included 415 pharmacies and 483 PMVs.

### Questionnaires

All questionnaires were developed in English. Final modifications were made to the questionnaires following an extensive pretesting exercise including pilot surveys .

The health facility provider questionnaire elicited information on the respondent’s background, family planning training experience, and barriers to family planning provision (Additional files 1, 2 and 3).

### Analysis

The software program, CsPro, was used for the data processing. The data entry, validation, and cleaning process involved double entry and verification/reconciliation. Data analysis was conducted in Stata Versions 12/13.

Barriers to contraceptives included in the analysis were age, parity, and marital status. Minimum age bias was defined as providers indicating the minimum age they would offer a method to a client as 15 years or older. Minimum parity included refusing to offer a method to clients based on any parity - including nulliparous clients. Marital status barrier was noted if providers indicated they would not offer a method to an unmarried individual. Service providers were only asked about the specific barriers for those methods that the provider indicated that he/she provide at their facility. The methods included in the survey were male and female condoms, combined and progestin-only oral contraceptive pills, emergency contraception, injectables, and IUD. Providers were also asked about barriers to implant, female sterilization, and male sterilization provision but there were too few providers responding to these questions to include these methods in the analysis. In the analyses, only male condoms were used for the condom category and only combined oral contraceptives were used for the oral contraceptive pill category. A bias score was calculated for each provider by method - one point assigned for each of the three barrier types if indicated by the provider for a possible range of 0–3. The bias score was then averaged across providers by method and summed overall.

All interviewed providers were asked about family planning training experience. For the providers at pharmacies and PMVs, family planning training experience was categorized as any or none. For providers at health facilities, the questions about training were specific to any experience of family planning in-service training, which is training that occurs after completion of a professional degree or specific training and is typically completed while employed.

### Ethical considerations

Study procedures, consent forms, and questionnaires used for the surveys were submitted and approved by the Nigerian Health Research Ethics Committee as well as the Institutional Review Board at the University of North Carolina at Chapel Hill.

## Results

Most health facility-based providers (60%) surveyed were some combination of nurse and/or midwife. The smallest proportion of the sample were doctors (6%), and the remaining were Community Health Extension Workers (CHEW) (32%) (see Table 1). Abuja and Benin City had the lowest prevalence of CHEWs in the sample at 26–29%, Zaria had the lowest proportion of Nurse/Midwives at 52%, and Ibadan and Kaduna had the lowest proportion of doctors at 2–3% (data not shown). Most providers at pharmacies were employees (65%) while a third were pharmacists. Similar to pharmacies, most PMV providers were employees (76%), while other common employee types were nurse/midwives (12%) and CHEWs (9%). The majority of the family planning providers in health facilities surveyed were female (89%) compared to 46% of pharmacists, and 37% of PMV providers. The average age of health facility providers for the full sample was 37 years. Most health facility providers in the full sample were Christian (69%); however, the providers were predominately Muslim in Ilorin and Zaria (data not shown).

**Table 1:** Health facility, pharmacist, and PMV family planning providers’ background characteristics, Nigeria 2010

	Health facility providers (*n* = 1,479)	Pharmacists (*n* = 415)	PMVs (*n* = 483)
Employee type	%	%	%
Doctor	5.8***	0.0	1.0
Nurse/Midwife	59.9	1.0	11.6
CHEW	32.3	0.2	9.3
Pharmacist	0.0	33.7	1.9
Other Employee	1.8	65.1	76.0
Missing	0.2	0.0	0.2
Sex			
Female	88.8***	46.0	37.3
Male	11.2	51.1	55.9
Missing	0.0	2.9	6.8
Age (mean)	37.0	m	m
Religion			
Christian	68.9	m	m
Islam/Other	31.1	m	m
City			
Abuja	12.8***	23.1	18.2
Benin City	15.9	19.5	19.5
Ibadan	16.2	22.2	17.2
Ilorin	18.5	11.6	13.7
Kaduna	24.3	18.8	16.4
Zaria	12.3	4.8	15.1
Any FP Training Experience			
No	59.5	58.3	54.9
Yes	40.5	41.7	45.1

*m* Data are not available

*p ≤ 0.05; **p ≤0.01; ***p ≤ 0.001; Chi Square tests of the association between demographic and provider type

Nearly a quarter of the health facility sample was from Kaduna (24%) while 12–13% were from Zaria and Abuja, respectively. Very few of the pharmacy sample was from Zaria (5%) and Ilorin (12%) and the largest share was from Abuja (23%) and Ibadan (22%). The largest proportion of the PMV sample was from Benin City (20%) and the smallest was from Ilorin (14%). The majority of health facility providers (60%) had no in-service family planning training experience while 42% of pharmacists and 45% of PMV operators had family planning training experience (see Table 1).

Health facility providers, pharmacists, and PMV operators reported restricting access to family planning methods based on demographic factors (see Table 2). Minimum age restrictions ranged between 70 and 93% across method and provider type. Restrictions based on minimum age were high for all methods and types of providers but were relatively lower for provision of condoms, EC, and pills (70–87%), and highest for injectables and IUDs from (84 to 93%). Health facility providers were less likely to have a minimum age bias for EC (70%) than pharmacists or PMVs (75–83%). In contrast, health facility providers had greater minimum age bias for pills (87%) than did pharmacists or PMVs (80–82%).

**Table 2:** Health facility, Pharmacy, and PMV family planning providers’ prevalence of restriction of clients’ access to contraceptive methods by restriction and method, Nigeria 2010

	Health facility providers		Pharmacists		PMV Operators	
Minimum age	n	%	n	%	n	%
Male condom	692	73.1	410	76.3	474	70.7
Pill	906	86.9**	318	81.8	313	79.9
EC	395	70.1***	292	82.9	183	75.4
Injectable	1071	88.5*	289	84.1	na	na
IUD	560	93.2	na	na	na	na
Minimum parity						
Male condom	692	3.0*	410	2.9	474	5.7
Pill	906	38.3***	318	28.9	313	43.5
EC	395	12.7*	292	12.3	183	20.2
Injectable	1071	64.7***	289	21.8	na	na
IUD	560	53.4	na	na	na	na
Marital status						
Male condom	692	10.4	410	9.5	474	7.2
Pill	906	51.3***	318	41.5	313	57.5
EC	395	16.7**	292	26.4	183	23.5
Injectable	1071	73.5***	289	45.3	na	na
IUD	560	67.3	na	na	na	na
Overall bias score						
Male condom	692	0.9	410	0.9	474	0.8
Pill	906	1.8***	318	1.5	313	1.8
EC	395	1.0***	292	1.2	183	1.2
Injectable	1071	2.3***	289	1.5	na	na
IUD	560	2.1	na	na	na	na
Total^a^		3.7		3.6		3.8

*na* Not applicable

*p ≤ 0.05; **p ≤0.01; ***p ≤ 0.001; Chi Square, Anova, and *t*-tests of the association between bias and provider type

^a^not including injectable or IUD

Minimum parity restrictions ranged between 3 and 65% across method and provider type. Restrictions based on minimum parity were lowest for provision of condoms (3–6%) followed by EC (12–20%). Among pharmacists only, pill use (29%) was more likely to be restricted by parity than injectable use (22%). Among health facility providers, injectables were the most likely to be restricted (65%) by parity - even more so than IUDs (53%). Nearly half of PMV operators (44%) indicated they restrict access to pills based upon parity compared to fewer pharmacists and health facility providers (29–38%). Injectable use was three times more likely to be restricted based upon parity among health facility providers (65%) than pharmacists (22%).

Marital status restrictions ranged between 7 and 74% across method and provider type. Restrictions based on marital status were lowest for provision of condoms (7–10%) and EC (17–26%), and highest for IUDs (67%) and injectables (45–73%). Nearly half of all provider types restrict access to pills based upon marital status (42–58%). As with minimum parity, health facility providers were more likely to restrict access to injectables (74%) than IUDs (67%) and were much more likely than pharmacists (45%) to restrict access based upon marital status.

For all health providers, minimum age bias was the most common bias while a minimum parity was the least common bias. Condoms consistently had the lowest overall bias score, followed by EC, pills, IUD, and injectables. Pharmacists had the overall bias score for both pills and injectables (1.5). Health facility providers had a higher bias towards injectables (2.3) as compared to IUDs (2.1). Health facility providers have a higher bias score for injectables (2.3) when compared to pharmacists (1.5). When comparing overall bias scores by method and provider type, PMV providers have the highest overall bias score for condoms, pills, and injectables combined (3.8) and pharmacists have the lowest (3.6) (see Table 2).

Provider biases in service provision were examined by experience of in-service family planning training and type of provider (see Table 3). Experience of in-service training was associated with a lower prevalence of marital status service provision biases for pill, injectable, and IUD methods among nurses/midwives and CHEWs. Training also affected the minimum parity bias for injectable provision among nurses/midwives as well as pill provision among CHEWs. The effect of training was in the opposite direction than expected for pill and injectable minimum age bias for both nurse/midwives and CHEWs. The overall bias score was lower for providers who received in-service training for pill, injectable, and IUDs among nurses/midwives and pills and IUDs among CHEWs as compared to those with no in-service training (Table 3). Unlike for the health facility providers, there was no, or negative effects, of family planning training experience on pharmacists and PMV operators’ biased service provision (see Table 4).

**Table 3:** Health facility family planning providers’ prevalence of restriction of clients’ access to contraceptive methods among Nurses/Midwives and CHEWs by restriction, method, and experience of in-service family planning training experience, Nigeria 2010

	Nurse/Midwife				CHEW			
	Any In-service FP Training		No In-service FP Training		Any In-service FP Training		No In-service FP Training	
Minimum age	n	%	n	%	n	%	n	%
Male condom	245	80.0	176	75.6	122	65.6	102	63.7
Pill	276	91.3*	246	85.8	145	89.7**	181	77.9
EC	156	72.4	104	64.4	44	77.3	48	70.8
Injectable	307	91.2*	320	86.3	155	92.9*	222	84.2
IUD	223	92.8	126	96.0	96	92.7	67	91.0
Minimum parity								
Male condom	245	3.7	176	3.4	122	1.6	102	2.9
Pill	276	33.3	246	39.4	145	29.7***	181	50.8
EC	156	10.3	104	13.5	44	13.6	48	20.8
Injectable	307	57.3**	320	67.5	155	65.2	222	72.5
IUD	223	48.4	126	58.7	96	51.0	67	64.2
Marital status								
Male condom	245	7.8	176	10.2	122	10.7	102	15.7
Pill	276	40.6***	246	56.5	145	39.3***	181	71.8
EC	156	14.1	104	16.4	44	15.9	48	22.9
Injecable	307	65.2***	320	78.1	155	67.7***	222	85.1
IUD	223	59.2***	126	81.0	96	61.5**	67	80.6
Bias score								
Male condom	245	0.9	176	0.9	122	0.8	102	0.8
Pill	276	1.7*	246	1.8	145	1.6***	181	2.0
EC	156	1.0	104	0.9	44	1.1	48	1.1
Injectable	307	2.1*	320	2.3	155	2.3	222	2.4
IUD	223	2.0***	126	2.4	96	2.1*	67	2.4
Total		7.7		8.3		7.9		8.7

*p ≤ 0.05; **p ≤0.01; ***p ≤ 0.001; Chi Square, Fisher’s Exact, and Anova tests of the association between bias and experience of training

**Table 4:** Pharmacy and PMV providers’ prevalence of restriction of clients’ access to contraceptive methods by restriction, method, and family planning training experience, Nigeria 2010

	Pharmacist				PMV			
	Any FP Training		No FP Training		Any FP Training		No FP Training	
Minimum age	n	%	n	%	n	%	n	%
Male condom	172	79.7	238	74.0	213	75.6*	261	66.7
Pill	148	89.2***	170	75.3	153	85.0*	160	75.0
EC	127	89.8**	165	77.6	89	82.0*	94	69.2
Injectable	130	88.5	159	80.5	na	na	na	na
Minimum parity								
Male condom	172	2.9	238	2.9	213	7.5	261	4.2
Pill	148	29.1	170	28.8	153	42.5	160	44.4
EC	127	11.8	165	12.7	89	25.8	94	14.9
Injectable	130	22.3	159	21.4	na	na	na	na
Marital status								
Male condom	172	7.6	238	10.9	213	6.6	261	7.7
Pill	148	43.9	170	39.4	153	61.4	160	53.8
EC	127	24.4	165	27.9	89	31.5*	94	16.0
Injectable	130	41.5	159	48.4	na	na	na	na
Bias score								
Male condom	172	0.9	238	0.9	213	0.9*	261	0.8
Pill	148	1.6	170	1.4	153	1.9	160	1.7
EC	127	1.3	165	1.2	89	1.4**	94	1.0
Injectable	130	1.5	159	1.5	na	na	na	na
Total^a^		3.8		3.5		4.2		3.5

*na* Provider doesn’t have jurisdiction to provide method

*p ≤ 0.05; **p ≤0.01; ***p ≤ 0.001; Chi Square, Fisher’s Exact, and t-tests of the association between bias and any family planning training experience

^a^not including injectable

## Discussion

Provider imposed eligibility barriers in terms of age, parity, and marital status in the six study cities in Nigeria were pervasive. Restrictions based on age, parity, and marital status, are not included as part of the medical recommendations for contraceptive provision by the World Health Organization [7] or by the Nigerian Federal Government [22].

The most prevalent restriction across method and provider type was minimum age. Age barriers have been identified as a prevalent barrier to contraceptive use in Kenya, Senegal, and Tanzania as well [14, 15, 17]. Research in Ghana has shown that provider fears of contraceptive induced infertility contribute to their age biases [16]. Fertility is incredibly important in Nigerian societal norms - infertility is associated with a life of no value [23]. Thus, providers fear that they will be accused of causing the infertility by the contraceptive user, herself, or her partner [16]. Increasing knowledge about the causes of infertility and reducing the myths about potential contribution of contraceptive use to infertility, among family planning providers and the general public, could potentially reduce age biases in contraceptive service provision.

Given the high prevalence of minimum age restrictions on contraceptive commodity provision in tandem with the rapid growth of youth in urban contexts, attention needs to be given to attitudes and perceptions of adolescent and young adult access to high quality reproductive health services. Other studies have examined interventions aimed at making reproductive health services adolescent friendly. While these studies have shown some positive effects of youth friendly service programs - the prior studies suggest that a lack of community support for adolescents accessing and using contraceptive services may be the biggest factor in preventing adolescents from accessing contraception [24, 25]. Therefore, a multifaceted approach - that targets community-level norms, not just the clinic and providers, will potentially have the most positive effects on adolescents’ unhindered access to contraceptives. Other studies have found effects of training of providers on knowledge, understanding of adolescent barriers to access, and some health provider attitudes toward provision of contraceptives to adolescents; however, even after adolescent targeted training, providers in one study remained reluctant to provide methods to adolescents based on age and parity factors [26].

In Tanzania, 20% of providers were found to restrict access to contraception based on marital status [15]. In our study in Nigeria the same was true for EC but fewer providers reported restricting access to male condoms based upon marital status and more did so for pills (42–58%), IUD (67%), and injectables (45–74%). This indicates that unmarried individuals might be able to access male condoms and EC - but have much lower chances of finding a provider willing to give them more effective user-controlled methods of pills, IUDs, or injectables. As a result, unmarried individuals might choose to not use contraceptives or they might opt for methods that they are more likely to be able to access but are less effective. Since pharmacists were less likely to bias against marital status in pill and injectable provision - one strategy would be to promote pharmacists as a source for pills and injectable use among unmarried individuals. Another strategy would be to find ways to work with providers of all types to recognize their marital status biases, show them the impact of these biases on individuals and communities, and find ways to creatively challenge these biases from within.

In this Nigerian setting, the most widely used method, the male condom, was also the method with the least amount of provider bias. The low level of provider bias (3–10%), other than for minimum age (71–76%), might be due to the gender of the individual accessing the method. If men are more likely to access male condoms than women - the providers might associate this method with the main user, and therefore, along with the social conventions about sexual behaviors - have less bias towards males using contraception who are young not married, and not with children, than they would have toward women. However, this is not what was observed in a simulated study of young male clients accessing contraception as compared to females in Uganda [19].

The second most commonly used method, the injectable, also has the highest level of bias - as has been reported in Ghana, as well [16]. From 84 to 89% of injectable providers restrict access based on a minimum age. Among health facility providers, the lowest prevalent provider bias for injectables was minimum parity - and that, the least common bias, was still as high as 65% of providers.

According to the NDHS, 60% of current modern method users obtained their contraceptives from private sources - most (38%) were obtained from PMVs. Just under a third (29%) of modern method users obtained methods from the public sector - mostly from government hospitals. PMVs supplied over half of the pills and male condoms among all current modern method users. Government hospitals provided the majority of IUDs and injectables for the nation [2]. Health facility providers were more biased in contraceptive provision as compared to PMVs and pharmacists. This is in juxtaposition to other studies in Tanzania that have found lower level staff have more conservative distribution attitudes [15].

Research in Tanzania found no effect of recent in-service training on provider barriers to family planning [15]. In our study, in-service family planning training reduced marital status bias among health facility providers for pills, injectables, and IUDs. The training had no, or the opposite, effect on minimum age bias. The effect of family planning training on pharmacists and PMVs was either non-existent or in the opposite direction of what would be expected. It is difficult to know why the family planning training experience for health facility providers was only partially effective and completely ineffective for pharmacists and PMVs due to the range of potential family planning training programs available in Nigeria. It is possible that the trainings focused more on the proper techniques for administering contraceptives, the limits of what each provider is legally able to do, and the medical eligibility criteria - as opposed to socially imposed medical barriers. Given the particular importance of PMVs in family planning service delivery in Nigeria, it would be imperative to find out what family planning training exists for PMVs and determine how it could be strengthened to address these providers’ medical barriers to provision.

Training programs must acknowledge and situate the information and skills provided within the cultural norms and attitudes that shape the social environment in which family planning service providers practice. Family planning training has been targeted to higher level providers -doctors are more likely to receive training than other tiers of providers [21]. Given how few doctors interact with family planning clients - efforts to increase family planning training experience among other types of providers, especially those most likely to interact with family planning clients - such as nurse/midwives, CHEWs, pharmacists, and PMVs, is imperative.

As has been noted by other researchers, family planning providers restrict access to contraceptive methods based on motivations to protect their clients, follow the social norms of their communities and themselves, or to protect themselves from blame from negative effects of contraceptive use [16]. It is important to note that provider restrictions in this setting likely come from a caring space - so eliminating provider imposed restrictions to contraceptive use must start from this framework. There are a number of strategies that have been noted in the literature to combat this issue, some of which include: rewriting guidelines - specifically to include direct guidance for providing contraceptives to traditionally under-served groups, such as adolescents; increased supervising - either through more frequent interaction, physically or via technology; training of supervisors and providers; as well as using videos of clients turned away from services desired and in-person client testimonies from populations often restricted, such as youth, during training events [27]. Researchers have called on more than training to address provider restrictions to family planning services. In particular, there is a call for a “performance improvement” approach that is more holistic than training on knowledge and skills, and includes a recognition of the constellation of factors that impact provider effectiveness, as well as the performance of the entire system that encompasses providers [2, 28].

There are a few limitations of this study. The providers were asked to self-report their responses to interviewers - so providers may or may not have reported their average practice accurately due to various factors, including social desirability bias. Also, providers were asked about service provision of implants, female sterilization, and male sterilization but too few providers in this sample were providing these methods at the time of the survey to include them in the analyses.

Future research on this topic could examine the effects of provider bias in contraceptive method provision on clients actual adoption and continuation of a method. Further, studies can examine how clients navigate provider barriers, the impact of pharmacist’s and PMV operator’s bias on referral patterns to service delivery sites that can offer longer term methods of contraception, and the effectiveness of improving training opportunities for all tiers of contraceptive providers on inclusive contraceptive provision.

## Conclusion

A constellation of creative interventions [29] aimed at reducing, and eventually eliminating, provider imposed restrictions to family planning use in urban Nigeria are needed urgently to make family planning truly accessible to all those Nigerians who desire to use contraceptives to plan their families.

## Additional files

Additional file 1: Providers interview FINAL FROZEN. Health Facility Providers Survey. (PDF 299 kb)

Additional file 2: Pharmacy Audit FINAL FROZEN. Pharmacists Survey. (PDF 226 kb)

Additional file 3: PMS FINAL FROZEN. PMV Operators Survey. (PDF 252 kb)

## Abbreviations

ANC: Antenatal care
CHEW: Community health extension worker
EC: Emergency contraception
FP: Family planning
IUD: Intrauterine device
LGA: Local government area
NDHS: Nigeria Demographic and Health Survey
PMV: Patent medical vendor
SDP: Service delivery points

## References

[1] Population Reference Bureau (2016). 2016 World Population Data Sheet.

[2] National Population Commission (NPC) [Nigeria] and ICF Macro (2009). Nigeria Demographic and Health Survey 2008.

[3] National Population Commission (NPC) [Nigeria] and ICF International (2014). Nigeria Demographic and Health Survey 2013.

[4] Measurement, Learning and Evaluation (MLE) Project (2012). 2010–2011 Baseline Household Survey Report for the Nigerian Urban Reproductive Health Initiative.

[5] RamaRao S, Lacuesta M, Costello M, Pangolibay B, Jones H (2003). The Link Between Quality of Care and Contraceptive Use. Int Fam Plan Perspect.

[6] Shelton JD (2001). The Provider Perspective: Human after All. Int Fam Plan Perspect.

[7] World Health Organization (2010). Medical eligibility criteria for contraceptive use.

[8] Bertrand JT, Hardee K, Magnani RJ, Angle MA (1995). Access, quality of care and medical barriers in family planning programs. Int Fam Plan Perspect.

[9] Shelton JD, Angle MA, Jacobstein RA (1992). Medical barriers to access to family planning. Lancet.

[10] Calhoun LM, Speizer IS, Rimal R, Sripad P, Chatterjee N, Achyut P, Nanda P (2013). Provider imposed restrictions to clients’ access to family planning in urban Uttar Pradesh, India: a mixed methods study. BMC Health Serv Res.

[11] Hebert LE, Schwandt HM, Boulay M, Skinner J (2013). Family planning providers’ perspectives on family planning service delivery in Ibadan and Kaduna, Nigeria: a qualitative study. J Fam Plann Reprod Health Care.

[12] Nalwadda G, Miremb F, Tumwesigye NM, Byamugisha J, Faxelid E (2011). Constraints and prospects for contraceptive service provision to young people in Uganda: providers’ perspectives. BMC Health Serv Res.

[13] Robinson R, Moshabela M, Owusu-Ansah L, Kapungu C, Geller S (2011). Barriers to Intrauterine Device Uptake in a Rural Setting in Ghana. Health Care Women Int.

[14] Sidze EM, Lardoux S, Speizer IS, Faye CM, Mutua MM (2014). Young Women’s Access to and Use of Contraceptives: The Role of Providers’ Restrictions in Urban Senegal. Int Perspect Sex Reprod Health.

[15] Speizer IS, Hotchkiss DR, Magnani RJ, Hubbard B, Nelson K (2000). Do Service Providers in Tanzania Unnecessarily Restrict Clients’ Access to Contraceptive Methods?. Int Fam Plan Perspect.

[16] Stanback J, Twum-Baah K (2001). Why do family planning providers restrict access to services? An examination in Ghana. Int Fam Plan Perspect.

[17] Tumlinson K, Okigbo CC, Speizer IS (2015). Provider barriers to family planning access in urban Kenya. Contraception.

[18] Wood K, Jewkes R (2006). Blood Blockages and Scolding Nurses: Barriers to Adolescent Contraceptive Use in South Africa. Reprod Health Matters.

[19] Nalwadda G, Tumwesigye NM, Faxelid E, Byamugisha J, Miremb F (2011). Quality of Care in Contraceptive Services Provided to Young People in Two Ugandan Districts: A Simulated Client Study. PLoS One.

[20] Kipp W, Chacko S, Laing L, Kabagambe G (2007). Adolescent reproductive health in Uganda: Issues related to access and quality of care. Int J Adolesc Med Health.

[21] Qureshi AM (2010). Case Study: Does training of private networks of family planning clinicians in urban Pakistan affect service utilization?. BMC Inter Health Hum Rights.

[22] Federal Ministry of Health, Nigeria (2009). National Family Planning/Reproductive Health Service Protocols.

[23] Koster-Oyekan W (1999). Infertility among Yoruba Women: Perceptions on Causes, Treatments and Consequences. Afr J Reprod Health.

[24] Mmari KN, Magnani RJ (2003). Does Making Clinic-based Reproductive Health Services More Youth-friendly Increase Service Use by Adolescents? Evidence From Lusaka, Zambia. J Adolesc Health.

[25] Tavrow P, Withers M, McMullen K (2012). Age matters: differential impact of service quality on contraceptive uptake among post-abortion clients in Kenya. Cult Health Sex: An Inter J Res Interv Care.

[26] Meuwissen LE, Gorter AC, Kester ADM, Knottnerus JA (2006). Can a comprehensive voucher programme prompt changes in doctors’ knowledge, attitudes and practices related to sexual and reproductive health care for adolescents? A case study from Latin America. Trop Med Int Health.

[27] Malarcher S (2010). Social determinants of sexual and reproductive health: informing future research and programme implementation.

[28] Stolovitch HD, Keeps EJ (1992). Handbook of Human Performance Technology.

[29] Vlassoff C, Fonn S (2001). Health Workers for Change as a health systems management and development tool. Health Policy Plan.

