# Supplementary material for: Contraceptive service provider imposed restrictions to contraceptive access in urban Nigeria
Source: BMC Health Serv Res. 2017 Apr 12;17:268. doi: 10.1186/s12913-017-2233-0 (PMC5389090; doi:10.1186/s12913-017-2233-0)
Supplement: Supplementary file 2 — Pharmacy Audit FINAL FROZEN. Pharmacists Survey. (PDF 226 kb) [file 12913_2017_2233_MOESM2_ESM.pdf]

MLE MEASUREMENT, LEARNING & EVALUATION PROJECT FOR THE URBAN REPRODUCTIVE HEALTH INITIATIVE

Measurement, Learning & Evaluation (MLE) Project
Pharmacy Audit – Nigeria - 2011

CITY NAME & CODE ______________________________ [__]
(Abuja=1, Benin=2, Ibadan=3, Ilorin=4, Kaduna=5, Zaria=6)

LGA NAME & CODE______________________________ [__|__|__]

LOCALITY NAME & CODE ______________________________ [__|__|__|__]

PHARMACY NAME AND CODE ______________________________ [__|__|__|__|__]

PHYSICAL ADDRESS OF THIS PHARMACY SHOP ______________________________

LOCATION OF PHARMACY
GPS Reading
Altitude .... [| | |]

Latitude . . . . N [|] . [| | | |]

Longitude . . . . E [| |] . [| | | |]

MANAGING AUTHORITY

Government...1	Mission..4
Private (for-profit)...2	Other ______________________6 (Specify)
NGO (not-for-profit)..3	

INTERVIEWER VISITS

**VISIT No.**	**1**	**2**	**3**	**FINAL VISIT**
DATE	DAY/ MONTH/YEAR [___/_____/_11_]	DAY/ MONTH/ YEAR [___/_____/__11_]	DAY/ MONTH/ YEAR [___/_____/__11_]	DAY [___|___] MONTH [___|___] YEAR [_2_|_0_|_1_|_1_]
INTERVIEWER'S NAME	____________	____________	____________	____________
INTERVIEWER CODE	[| |]	[| |]	[| |]	[| |]
RESULT*	[ ]	[ ]	[ ]	[ ]
**NEXT VISIT: DATE**	[___/_____/11_]	[___/_____/11_]	[___/_____/11_]	TOTAL NO. OF VISITS
**TIME**	[|] [|] H H M M	[|] [|] H H M M	[|] [|] H H M M	[ ]

***RESULT CODES:**
1. COMPLETED
2. PHARMACY MOVED OR IS DESTROYED
3. RESPONDENT NOT AVAILABLE
4. RESPONDENT REFUSED
5. PARTLY COMPLETED
6. POSTPONED
7. OTHER ______________________ (Specify)

	ENGLISH	HAUSA	YORUBA	IGBO	PIDGIN	OTHER(SPECIFY)	TRANSLATOR USED? YES	NO
LANGUAGE OF INTERVIEW	1	2	3	4	5	6__________	YES	NO
NATIVE LANGUAGE OF RESPONDENT	1	2	3	4	5	6__________	1	2

FIND THE PERSON IN CHARGE OF MEDICINES. IF HE/SHE IS NOT PRESENT, ASK TO SEE THE PROVIDER MOST KNOWLEDGEABLE ABOUT PHARMACEUTICAL PROCEDURES, PURCHASING, AND MANAGEMENT	
POSITION OF PERSON INTERVIEWED	SEX OF PERSON INTERVIEWED
NON-PHARMACIST MANAGER/PROPRIETOR.....1<br>PHARMACIST MANAGER/PROPRIETOR……,,,,...2<br>PHARMACIST…………………………………..……..3<br>PHARMACY TECHNICIAN…………………..……..4<br>ATTENDANTS………………………………………..5<br>OTHER ________________________________6<br>(SPECIFY)	MALE……………………………………..1<br>FEMALE……………………………….…2

SUPERVISOR	OFFICE EDITOR	KEYED BY
NAME………………………………………………….<br>CODE: [ ][ ][ ]<br>DATE [__/___/ 11_]<br>DD MM YY	NAME………………………………………………….<br>CODE: [ ][ ][ ]<br>DATE [__/___/ 11_]<br>DD MM YY	NAME…………………………………………………………………….<br>CODE: [ ][ ][ ]<br>DATE [__/__/ 11_]<br>DD MM YY

Q1.	RECORD THE TIME (IN 24 HOUR FORMAT)	Hour ………….[ ][ ] Minutes ………..[ ][ ]	
**GENERAL FACILITY INFORMATION**			
**Source**	**Questions**	**Coding**	**Skip**
Q2.	In this PHARMACY shop, how many regular, permanent staff (workers) work here?	[ ][ ]	
Q3.	In what year did this facility open?<br><br>PROBE: This is very important. Can you tell me how old this facility is? For example, would you say it is about 1, 2, 3, 7, 11, etc years old? **FILL IN EITHER YEAR OPENED OR YEARS OLD.**	YEAR OPENED……[ ][ ][ ][ ]<br>DON'T KNOW……9998<br>**OR**<br>YEARS OLD . . . . . .[ ][ ]	
Q4.	On average, how many hours per day is the pharmacy open?	HOURS PER DAY . . . .[ ][ ]	
Q5.	On average, how many days per week is the facility open?	DAYS PER WEEK . . . .[ ]	
Q6.	Is there a trained registered pharmacist who works at least part-time here?	YES……………..…. . . . . 1<br>NO . . . . 2 →	**Q8**
Q7.	How many hours per week does the trained registered pharmacist work here?	Hours per week . . . . [ ][ ][ ]	
Q8.	Who is the principal person responsible for managing medical supplies at this pharmacy? By this I mean the person responsible for ordering, receiving and controlling medical supplies.	PHARMACIST . . . . 1<br>DISPENSER . . . . 2<br>NON-PHARMACIST MANAGER...…….3<br>NON-PHARMACIST PROPRIETOR…..4<br>SUPPLIES OFFICER . . . . .5<br>STORE ASSISTANT . . . . .7<br>OTHER _____________________ 6<br>(SPECIFY)	
Q9.	Is there a stock register where the amount of each medicine received, the amount disbursed, and the amount present today (stock balance) is recorded?	YES, OBSERVED . . . . 1<br>YES, REPORTED, NOT SEEN . . . 2<br>NO . . . . 3 →	**Q12**
Q10.	How often do you update or reconcile your inventory/stock records?	EVERY [ ][ ] DAY(S)<br><br>THE DAY ITEMS ARE RECEIVED OR DISBURSED . ….… . . 95<br>NEVER . . . . 97<br>OTHER________________ (specify)..96	
Q11.	Is the stock maintenance system computerized?	YES . . . . 1<br>NO 2	

Q12.	**CIRCLE THE RESPONSE THAT BEST DESCRIBES THE SYSTEM.**	STOCK RECORDS UPDATED ON THE DAY ITEM RECEIVED/DISBURSED……………………1<br><br>STOCK RECORDS NOT ALWAYS UPDATED WHEN ITEM DISBURSED, BUT RECORD OF ITEMS RECEIVED /DISTRIBUTED OBSERVED…………………………………………2<br><br>NO RECORDS OBSERVED……………….....……3<br><br>RECORDS NOT UP TO DATE…………………..…4<br><br>OTHER______________________________6<br>(SPECIFY)	
Q13.	Have you received any training on family planning?	YES . . . . .1<br>NO . . . . .2<br>DON'T KNOW 8	**Q16**
Q14.	When was the last family planning training that you attended?	DAYS AGO…….1<br>WEEKS AGO…..2<br>MONTHS AGO…3<br>YEARS AGO……4<br>|__|__|	
Q15.	What were the issues covered in the last training?<br><br>**CIRCLE ALL MENTIONED SPONTANEOUSLY.**<br>**DO NOT READ OUT OPTIONS**	CONTRACEPTIVE TECHNOLOGY UPDATE…A<br>EXCLUSIVE BREASTFEEDING COUNSELING/LAM……………………………..B<br>NATURAL FP (STANDARD DAYS, CYCLE BEADS, ETC.)……………………………………C<br>FP COUNSELING SKILLS……………………D<br>PILLS…………………………………………....E<br>CONDOMS………………………………………...F<br>SPERMICIDE…………………………………….G<br>EMERGENCY CONTRACEPTIVE………..……H<br>IUD……………………………………………………I<br>INJECTABLES………………………………….J<br>DIAPHRAGM……………………………………..K<br>OTHERS ___________________________X<br>(SPECIFY)	
Q16.	Is information and counseling related to family planning ever provided by staff from this facility to clients?	YES... . ……………………………………..1<br>NO . . . . .2<br>DON'T KNOW 8	
Q17.	Before buying a method of family planning in this pharmacy, would you say that a woman receives FP information and counseling always, sometimes, or never?	YES, ALWAYS... . ………….…………..1<br>SOMETIMES . . . . .2<br>NEVER…………………………………...3<br>DON'T KNOW . . . .….8	
Q18.	OBSERVE WHETHER THERE ARE ANY FAMILY PLANNING PROMOTIONAL MATERIALS ON DISPLAY (EG, POSTERS, BROCHURES, DANGLERS, CALENDARS, ETC.)	DISPLAYED……………………….1<br>NOT DISPLAYED…………………2	
Q19.	Does this pharmacy provide family planning methods?	YES . . . . ………... .1 →<br>NO . . . . .2<br>DON'T KNOW 8	**Q22a**
Q20.	Would you be willing to sell family planning methods at this shop?	YES………………………………… .1<br>NO . . . . .2 →<br>DON'T KNOW . . . . .8 →	**END**<br>**END**
Q21.	Which methods would you be willing to sell?<br><br>**MULTIPLE RESPONSES POSSIBLE..**<br>**CIRCLE ALL MENTIONED.**	COMBINED PILL . . . . ……….A<br>PROGESTIN-ONLY PILL . …….………B<br>PILL (TYPE UNSPECIFIED) ..………...C<br>MALE CONDOM . . . . ……….…… D<br>FEMALE CONDOM . .. . ……….…...E<br>IUD……….... . . . . …. …………….F<br>SPERMICIDE …………..…………….....G<br>DIAPHRAGM . . . .……………H<br>INJECTABLES.. . . . . ……….…..I<br>IMPLANT . . . .…………J<br>EMERGENCY CONTRACEPTIVES . ...K<br>OTHER (specify)___________________X	**ALL GO TO END**

**ASK IF THE FOLLOWING CONTRACEPTIVES ARE AVAILABLE. FOR EACH ITEM, CIRCLE THE APPROPRIATE CODE.<br>FOR ALL ITEMS THAT ARE OBSERVED, ASK IF THERE HAS BEEN ANY STOCK-OUT (BRAND NOT AVAILABLE FOR AT LEAST 24 HOURS) DURING THE LAST 12 MONTHS AND LAST 30 DAYS.**									
CONTRA-CEPTIVE<br><br>(ASK FOR MOST POPULAR BRANDS)	Q22a. Does this pharmacy usually sell the following FP methods?	Q22b. What brands do you usually stock? **LIST ALL BRAND NAMES USUALLY STOCKED, EVEN IF CURRENTLY OUT OF STOCK. IF MORE THAN 3 BRANDS, LIST THE 3 MOST POPULAR BRANDS** (CODING TO BE DONE IN OFFICE).	Q22c. What is the retail price (in Naira) for ______ [PRODUCT/ BRAND]?	Q22d. What is the average retail sales volume in a month?	Q22e. Is ________ [PRODUCT/ BRAND] currently available?	Q22f. Has ______ [PRODUCT/ BRAND] been stocked out in this store for at least 24 hours in the last **ONE** year? **IF NO OR DON'T KNOW, SKIP TO NEXT BRAND.**	Q22g. In the past one year, for how many total days were you stocked out of ________ [PRODUCT/ BRAND] (all stock-outs combined)?	Q22h. Has ____ [PRODUCT/ BRAND] been stocked out for at least 24 hours in the last **ONE** month (30 days)? **IF NO OR DON'T KNOW, SKIP TO NEXT BRAND/ METHOD.**	Q22i. In the last 30 days, for how many total days were you stocked out of _____ [PRODUCT/ BRAND] (all stock-outs combined)?
(1) Combin-ation oral contra-ceptives (estrogen and pro-gestin)	YES..1<br>NO…2→**(2)**		RETAIL PRICE PER CYCLE:	SALES VOLUME (CYCLES):	YES . .1<br>NO …..2	YES….1<br>NO …..2<br>DK…….8	RECORD DAYS:	YES….1<br>NO …..2<br>DK…….8	RECORD DAYS:
		____________[__|__|__]<br>BRAND (1)	[__|__|__|__]<br>BRAND (1)	[__|__|__|__]<br>BRAND (1)	☐<br>BRAND (1)	☐<br>BRAND (1)	☐☐☐<br>BRAND (1)<br>Don't know…998	☐<br>BRAND (1)	☐☐<br>BRAND (1)<br>Don't know…98
		____________[__|__|__]<br>BRAND (2)	[__|__|__|__]<br>BRAND (2)	[__|__|__|__]<br>BRAND (2)	☐<br>BRAND (2)	☐<br>BRAND (2)	☐☐☐<br>BRAND (2)<br>Don't know…998	☐<br>BRAND (2)	☐☐<br>BRAND (2)<br>Don't know…98
		____________[__|__|__]<br>BRAND (3)	[__|__|__|__]<br>BRAND (3)	[__|__|__|__]<br>BRAND (3)	☐<br>BRAND (3)	☐<br>BRAND (3)	☐☐☐<br>BRAND (3)<br>Don't know…998	☐<br>BRAND (3)	☐☐<br>BRAND (3)<br>Don't know…98
(2) Progestin-only oral contra-ceptives	YES..1<br>NO…2□(3)		RETAIL PRICE PER CYCLE:	SALES VOLUME (CYCLES):	YES . .1<br>NO …..2	YES….1<br>NO …..2<br>DK…….8	RECORD DAYS:	YES….1<br>NO …..2<br>DK…….8	RECORD DAYS:
		____________[__|__|__]<br>BRAND (1)	[__|__|__|__]<br>BRAND (1)	[__|__|__|__]<br>BRAND (1)	☐<br>BRAND (1)	☐<br>BRAND (1)	☐☐☐<br>BRAND (1)<br>Don't know…998	☐<br>BRAND (1)	☐☐<br>BRAND (1)<br>Don't know…98
		____________[__|__|__]<br>BRAND (2)	[__|__|__|__]<br>BRAND (2)	[__|__|__|__]<br>BRAND (2)	☐<br>BRAND (2)	☐<br>BRAND (2)	☐☐☐<br>BRAND (2)<br>Don't know…998	☐<br>BRAND (2)	☐☐<br>BRAND (2)<br>Don't know…98
		____________[__|__|__]<br>BRAND (3)	[__|__|__|__]<br>BRAND (3)	[__|__|__|__]<br>BRAND (3)	☐<br>BRAND (3)	☐<br>BRAND (3)	☐☐☐<br>BRAND (3)<br>Don't know…998	☐<br>BRAND (3)	☐☐<br>BRAND (3)<br>Don't know…98

CONTRA-CEPTIVE<br><br>(ASK FOR MOST POPULAR BRANDS)	Q22a. Does this pharmacy usually sell the following FP methods?	Q22b. What brands do you usually stock?<br>**LIST ALL BRAND NAMES USUALLY STOCKED, EVEN IF CURRENTLY OUT OF STOCK. IF THERE ARE MORE THAN 3 BRANDS, LIST THE 3 MOST POPULAR BRANDS** (CODING WILL BE DONE IN THE OFFICE).	Q22c. What is the retail price (in Naira) for ______ [PRODUCT/ BRAND]?	Q22d. What is the average retail sales volume in a month?	Q22e. Is ________ [PRODUCT/ BRAND] currently available?	Q22f. Has ______ [PRODUCT/ BRAND] been stocked out in this store for at least 24 hours in the last **ONE** year?<br>**IF NO OR DON'T KNOW, SKIP TO NEXT BRAND.**	Q22g. In the past one year, for how many total days were you stocked out of ________ [PRODUCT/ BRAND] (all stock-outs combined)?	Q22h. Has ____ [PRODUCT/ BRAND] been stocked out for at least 24 hours in the last **ONE** month (30 days)?<br>**IF NO OR DON'T KNOW, SKIP TO NEXT BRAND/ METHOD.**	Q22i. In the last 30 days, for how many total days were you stocked out of _____ [PRODUCT/ BRAND] (all stock-outs combined)?
(3) Emergency contraceptives	YES..1<br>NO…2→**(4)**	____________[__|__|__]<br>BRAND (1)<br><br>____________[__|__|__]<br>BRAND (2)<br><br>____________[__|__|__]<br>BRAND (3)	RETAIL PRICE PER PACK:<br><br>[__|__|__|__|__]<br>BRAND (1)<br><br>[__|__|__|__|__]<br>BRAND (2)<br><br>[__|__|__|__|__]<br>BRAND (3)	SALES VOLUME (PACKS):<br><br>[__|__|__|__|__]<br>BRAND (1)<br><br>[__|__|__|__|__]<br>BRAND (2)<br><br>[__|__|__|__|__]<br>BRAND (3)	YES . .1<br>NO …..2<br><br>BRAND (1)<br><br>BRAND (2)<br><br>BRAND (3)	YES….1<br>NO …..2<br>DK…….8<br><br>BRAND (1)<br><br>BRAND (2)<br><br>BRAND (3)	RECORD DAYS:<br><br>BRAND (1)<br>Don't know…998<br><br>BRAND (2)<br>Don't know…998<br><br>BRAND (3)<br>Don't know…998	YES….1<br>NO …..2<br>DK…….8<br><br>BRAND (1)<br><br>BRAND (2)<br><br>BRAND (3)	RECORD DAYS:<br><br>BRAND (1)<br>Don't know…98<br><br>BRAND (2)<br>Don't know…98<br><br>BRAND (3)<br>Don't know…98
(4) Male condoms	YES..1<br>NO…2→**(5)**	____________[__|__|__]<br>BRAND (1)<br><br>____________[__|__|__]<br>BRAND (2)<br><br>____________[__|__|__]<br>BRAND (3)	RETAIL PRICE PER PIECE:<br><br>[__|__|__|__|__]<br>BRAND (1)<br><br>[__|__|__|__|__]<br>BRAND (2)<br><br>[__|__|__|__|__]<br>BRAND (3)	SALES VOLUME (PIECES):<br><br>[__|__|__|__|__]<br>BRAND (1)<br><br>[__|__|__|__|__]<br>BRAND (2)<br><br>[__|__|__|__|__]<br>BRAND (3)	YES . .1<br>NO …..2<br><br>BRAND (1)<br><br>BRAND (2)<br><br>BRAND (3)	YES….1<br>NO …..2<br>DK…….8<br><br>BRAND (1)<br><br>BRAND (2)<br><br>BRAND (3)	RECORD DAYS:<br><br>BRAND (1)<br>Don't know…998<br><br>BRAND (2)<br>Don't know…998<br><br>BRAND (3)<br>Don't know…998	YES….1<br>NO …..2<br>DK…….8<br><br>BRAND (1)<br><br>BRAND (2)<br><br>BRAND (3)	RECORD DAYS:<br><br>BRAND (1)<br>Don't know…98<br><br>BRAND (2)<br>Don't know…98<br><br>BRAND (3)<br>Don't know…98

CONTRA-CEPTIVE<br><br>(ASK FOR MOST POPULAR BRANDS)	Q22a. Does this pharmacy usually sell the following FP methods?	Q22b. What brands do you usually stock?<br>**LIST ALL BRAND NAMES USUALLY STOCKED, EVEN IF CURRENTLY OUT OF STOCK. IF THERE ARE MORE THAN 3 BRANDS, LIST THE 3 MOST POPULAR BRANDS** (CODING WILL BE DONE IN THE OFFICE).	Q22c. What is the retail price (in Naira) for ______ [PRODUCT/ BRAND]?	Q22d. What is the average retail sales volume in a month?	Q22e. Is ________ [PRODUCT/ BRAND] currently available?	Q22f. Has ______ [PRODUCT/ BRAND] been stocked out in this store for at least 24 hours in the last **ONE** year?<br>**IF NO OR DON'T KNOW, SKIP TO NEXT BRAND.**	Q22g. In the past one year, for how many total days were you stocked out of ________ [PRODUCT/ BRAND] (all stock-outs combined)?	Q22h. Has ____ [PRODUCT/ BRAND] been stocked out for at least 24 hours in the last **ONE** month (30 days)?<br>**IF NO OR DON'T KNOW, SKIP TO NEXT BRAND/ METHOD.**	Q22i. In the last 30 days, for how many total days were you stocked out of _____ [PRODUCT/ BRAND] (all stock-outs combined)?
(5) Female condoms	YES..1<br>NO…2→**(6)**	_____________[__|__|__]<br>BRAND (1)<br><br>_____________[__|__|__]<br>BRAND (2)<br><br>_____________[__|__|__]<br>BRAND (3)	RETAIL PRICE PER PIECE:<br><br>[__|__|__|__|__]<br>BRAND (1)<br><br>[__|__|__|__|__]<br>BRAND (2)<br><br>[__|__|__|__|__]<br>BRAND (3)	SALES VOLUME (PIECES):<br><br>[__|__|__|__|__]<br>BRAND (1)<br><br>[__|__|__|__|__]<br>BRAND (2)<br><br>[__|__|__|__|__]<br>BRAND (3)	YES . .1<br>NO …..2<br><br>☐<br>BRAND (1)<br><br>☐<br>BRAND (2)<br><br>☐<br>BRAND (3)	YES….1<br>NO …..2<br>DK…….8<br><br>☐<br>BRAND (1)<br><br>☐<br>BRAND (2)<br><br>☐<br>BRAND (3)	RECORD DAYS:<br><br>☐☐☐<br>BRAND (1)<br>Don't know…998<br><br>☐☐☐<br>BRAND (2)<br>Don't know…998<br><br>☐☐☐<br>BRAND (3)<br>Don't know…998	YES….1<br>NO …..2<br>DK…….8<br><br>☐<br>BRAND (1)<br><br>☐<br>BRAND (2)<br><br>☐<br>BRAND (3)	RECORD DAYS:<br><br>☐☐<br>BRAND (1)<br>Don't know…98<br><br>☐☐<br>BRAND (2)<br>Don't know…98<br><br>☐☐<br>BRAND (3)<br>Don't know…98
(6) Spermicide (foam, foaming tablets, gel)	YES..1<br>NO…2→**(7)**	_____________[__|__|__]<br>BRAND (1)<br><br>_____________[__|__|__]<br>BRAND (2)<br><br>_____________[__|__|__]<br>BRAND (3)	RETAIL PRICE PER UNIT:<br><br>[__|__|__|__|__]<br>BRAND (1)<br><br>[__|__|__|__|__]<br>BRAND (2)<br><br>[__|__|__|__|__]<br>BRAND (3)	SALES VOLUME (UNITS):<br><br>[__|__|__|__|__]<br>BRAND (1)<br><br>[__|__|__|__|__]<br>BRAND (2)<br><br>[__|__|__|__|__]<br>BRAND (3)	YES . .1<br>NO …..2<br><br>☐<br>BRAND (1)<br><br>☐<br>BRAND (2)<br><br>☐<br>BRAND (3)	YES….1<br>NO …..2<br>DK…….8<br><br>☐<br>BRAND (1)<br><br>☐<br>BRAND (2)<br><br>☐<br>BRAND (3)	RECORD DAYS:<br><br>☐☐☐<br>BRAND (1)<br>Don't know…998<br><br>☐☐☐<br>BRAND (2)<br>Don't know…998<br><br>☐☐☐<br>BRAND (3)<br>Don't know…998	YES….1<br>NO …..2<br>DK…….8<br><br>☐<br>BRAND (1)<br><br>☐<br>BRAND (2)<br><br>☐<br>BRAND (3)	RECORD DAYS:<br><br>☐☐<br>BRAND (1)<br>Don't know…98<br><br>☐☐<br>BRAND (2)<br>Don't know…98<br><br>☐☐<br>BRAND (3)<br>Don't know…98

CONTRA-CEPTIVE<br><br>(ASK FOR MOST POPULAR BRANDS)	Q22a. Does this pharmacy usually sell the following FP methods?	Q22b. What brands do you usually stock? **LIST ALL BRAND NAMES USUALLY STOCKED, EVEN IF CURRENTLY OUT OF STOCK. IF THERE ARE MORE THAN 3 BRANDS, LIST THE 3 MOST POPULAR BRANDS** (CODING WILL BE DONE IN THE OFFICE).	Q22c. What is the retail price (in Naira) for ______ [PRODUCT/ BRAND]?	Q22d. What is the average retail sales volume in a month?	Q22e. Is ________ [PRODUCT/ BRAND] currently available?	Q22f. Has ______ [PRODUCT/ BRAND] been stocked out in this store for at least 24 hours in the last **ONE** year? **IF NO OR DON'T KNOW, SKIP TO NEXT BRAND.**	Q22g. In the past one year, for how many total days were you stocked out of ________ [PRODUCT/ BRAND] (all stock-outs combined)?	Q22h. Has ____ [PRODUCT/ BRAND] been stocked out for at least 24 hours in the last **ONE** month (30 days)? **IF NO OR DON'T KNOW, SKIP TO NEXT BRAND/ METHOD.**	Q22i. In the last 30 days, for how many total days were you stocked out of _____ [PRODUCT/ BRAND] (all stock-outs combined)?
(7) Injectables (Depo, Noristerat)	YES..1<br>NO…2→**(8)**	____________[__|__|__]<br>BRAND (1)<br><br>____________[__|__|__]<br>BRAND (2)<br><br>____________[__|__|__]<br>BRAND (3)	RETAIL PRICE PER INJECTABLE:<br><br>[__|__|__|__]<br>BRAND (1)<br><br>[__|__|__|__]<br>BRAND (2)<br><br>[__|__|__|__]<br>BRAND (3)	SALES VOLUME (INJECTS)<br><br>[__|__|__|__]<br>BRAND (1)<br><br>[__|__|__|__]<br>BRAND (2)<br><br>[__|__|__|__]<br>BRAND (3)	YES . .1<br>NO …..2<br><br>BRAND (1)<br><br>BRAND (2)<br><br>BRAND (3)	YES….1<br>NO …..2<br>DK…….8<br><br>BRAND (1)<br><br>BRAND (2)<br><br>BRAND (3)	RECORD DAYS:<br><br>BRAND (1)<br>Don't know…998<br><br>BRAND (2)<br>Don't know…998<br><br>BRAND (3)<br>Don't know…998	YES….1<br>NO …..2<br>DK…….8<br><br>BRAND (1)<br><br>BRAND (2)<br><br>BRAND (3)	RECORD DAYS:<br><br>BRAND (1)<br>Don't know…98<br><br>BRAND (2)<br>Don't know…98<br><br>BRAND (3)<br>Don't know…98
(8) Implant (e.g. Implanon or Jadelle)	YES..1<br>NO…2→ **(Q23a)**	____________[__|__|__]<br>BRAND (1)<br><br>____________[__|__|__]<br>BRAND (2)<br><br>____________[__|__|__]<br>BRAND (3)	RETAIL PRICE PER IMPLANT:<br><br>[__|__|__|__]<br>BRAND (1)<br><br>[__|__|__|__]<br>BRAND (2)<br><br>[__|__|__|__]<br>BRAND (3)	SALES VOLUME (IMPLANTS):<br><br>[__|__|__|__]<br>BRAND (1)<br><br>[__|__|__|__]<br>BRAND (2)<br><br>[__|__|__|__]<br>BRAND (3)	YES . .1<br>NO …..2<br><br>BRAND (1)<br><br>BRAND (2)<br><br>BRAND (3)	YES….1<br>NO …..2<br>DK…….8<br><br>BRAND (1)<br><br>BRAND (2)<br><br>BRAND (3)	RECORD DAYS:<br><br>BRAND (1)<br>Don't know…998<br><br>BRAND (2)<br>Don't know…998<br><br>BRAND (3)<br>Don't know…998	YES….1<br>NO …..2<br>DK…….8<br><br>BRAND (1)<br><br>BRAND (2)<br><br>BRAND (3)	RECORD DAYS:<br><br>BRAND (1)<br>Don't know…98<br><br>BRAND (2)<br>Don't know…98<br><br>BRAND (3)<br>Don't know…98

Now I would like to ask you about your specific stocks of different family planning methods/products. **ONLY ASK ABOUT THOSE METHODS THAT ARE AVAILABLE FROM Q22a.**		
CONTRACEPTIVE	Q23a. Where does your stock of CONTRACEPTIVE (most popular brands) come from? CHOOSE ALL.	Q23b. On average, how long does it take to receive your supplies after you have placed an order? READ LIST.
(01) Combination oral contraceptives (estrogen and progestin)	Government…………………………… A<br>Intl NGO …………………………….… B<br>Local NGO……………………………..…C<br>Pharmacy wholesaler/distributor……...D<br>Other_______________________X<br>(Specify)<br>Don't know……………………………..…Z	One week or less…....1<br>Between 2-4 weeks….2<br>Between 5-8 weeks….3<br>More than 8 weeks…..4<br>Other __________6<br>(Specify)<br>Don't know . ………….8
(02) Progestin-only oral contraceptives	Government…………………………… A<br>Intl NGO …………………………….… B<br>Local NGO……………………………..…C<br>Pharmacy wholesaler/distributor……...D<br>Other_______________________X<br>(Specify)<br>Don't know……………………………..…Z	One week or less…....1<br>Between 2-4 weeks….2<br>Between 5-8 weeks….3<br>More than 8 weeks…..4<br>Other __________6<br>(Specify)<br>Don't know . ………….8
(03) Emergency contraceptives	Government…………………………… A<br>Intl NGO …………………………….… B<br>Local NGO……………………………..…C<br>Pharmacy wholesaler/distributor……...D<br>Other_______________________X<br>(Specify)<br>Don't know……………………………..…Z	One week or less…....1<br>Between 2-4 weeks….2<br>Between 5-8 weeks….3<br>More than 8 weeks…..4<br>Other __________6<br>(Specify)<br>Don't know . ………….8
(04) Male condoms	Government…………………………… A<br>Intl NGO …………………………….… B<br>Local NGO……………………………..…C<br>Pharmacy wholesaler/distributor……...D<br>Other_______________________X<br>(Specify)<br>Don't know……………………………..…Z	One week or less…....1<br>Between 2-4 weeks….2<br>Between 5-8 weeks….3<br>More than 8 weeks…..4<br>Other __________6<br>(Specify)<br>Don't know . ………….8
(05) Female condoms	Government…………………………… A<br>Intl NGO …………………………….… B<br>Local NGO……………………………..…C<br>Pharmacy wholesaler/distributor……...D<br>Other_______________________X<br>(Specify)<br>Don't know……………………………..…Z	One week or less…....1<br>Between 2-4 weeks….2<br>Between 5-8 weeks….3<br>More than 8 weeks…..4<br>Other __________6<br>(Specify)<br>Don't know . ………….8
(06) Spermicide	Government…………………………… A<br>Intl NGO …………………………….… B<br>Local NGO……………………………..…C<br>Pharmacy wholesaler/distributor……...D<br>Other_______________________X<br>(Specify)<br>Don't know……………………………..…Z	One week or less…....1<br>Between 2-4 weeks….2<br>Between 5-8 weeks….3<br>More than 8 weeks…..4<br>Other __________6<br>(Specify)<br>Don't know . ………….8
(07) Injectables (e.g., Depo Provera, Noristerat)	Government…………………………… A<br>Intl NGO …………………………….… B<br>Local NGO……………………………..…C<br>Pharmacy wholesaler/distributor……...D<br>Other_______________________X<br>(Specify)<br>Don't know……………………………..…Z	One week or less…....1<br>Between 2-4 weeks….2<br>Between 5-8 weeks….3<br>More than 8 weeks…..4<br>Other __________6<br>(Specify)<br>Don't know . ………….8
(08) Implant (Norplant)	Government…………………………… A<br>Intl NGO …………………………….… B<br>Local NGO……………………………..…C<br>Pharmacy wholesaler/distributor……...D<br>Other_______________________X<br>(Specify)<br>Don't know……………………………..…Z	One week or less…....1<br>Between 2-4 weeks….2<br>Between 5-8 weeks….3<br>More than 8 weeks…..4<br>Other __________6<br>(Specify)<br>Don't know . ………….8

Q24.	If there is a shortage of a specific CONTRACEPTIVE between routine orders, what is the most common procedure followed by this pharmacy?<br><br>- Submit special order to normal supplier<br><br>- Pharmacy purchases from private market<br><br>- Clients must purchase from another outlet<br><br>- Facility borrows from neighboring Pharmacy<br><br>- None of the above	SPECIAL ORDER . . . .1 →<br>PHARMACY PURCHASE . . . . 2 →<br>CLIENTS PURCHASE ELSEWHERE. ...3 →<br>PHARMACY BORROWS . . . . .4<br>NONE OF THE ABOVE . . . .5 →	**Q26**<br>**Q26**<br>**Q26**<br><br>**Q26**
Q25.	When you borrow supplies, from what outlet do you most often borrow?	NAME: ____________________________________	
Q26.	From which type of outlet do you borrow supplies?	Government……………………………..……………..1<br>Private (for-profit)…………………………….………2<br>NGO (not-for profit)…..…………………………..………3<br>Mission………………..…………………………..…………4<br>Other ___________________________________6<br>(Specify)	

ASK ONLY ABOUT THE FAMILY PLANNING METHODS THE PHARMACY IS PROVIDING IN Q22a. *Now I would like to ask you specifically about the contraceptive methods that you provide.*							
CONTRACEPTIVE	Q27a. What is the minimum age that you would offer this METHOD?	Q27b. What is the maximum age that you would offer this METHOD?	Q27c. Is there a minimum number of children a person must have before you will offer METHOD?	Q27d. What is that minimum number of children?	Q27e. Do you require a partner's consent before you will provide METHOD?	Q27f. Would you offer METHOD to an unmarried person?	Q27g. Do you require a prescription for a client to receive this METHOD?
(1) Combination oral contraceptives (estrogen and progestin)	[ ][ ]<br>NO MIN….93<br>DK……….98	[ ][ ]<br>NO MAX...93<br>DK……….98	YES . . .1<br>NO . . . .2 → **Q27e**<br>DK…….8 → **Q27e**	[ ][ ]	YES . . . .1<br>NO 2	YES . . . .1<br>NO 2	YES . . . .1<br>NO 2
(2) Progestin-only oral contraceptives	[ ][ ]<br>NO MIN….93<br>DK……….98	[ ][ ]<br>NO MAX...93<br>DK……….98	YES . . .1<br>NO . . . .2 → **Q27e**<br>DK…….8 → **Q27e**	[ ][ ]	YES . . . .1<br>NO 2	YES . . . .1<br>NO 2	YES . . . .1<br>NO 2
(3) Emergency contraceptives	[ ][ ]<br>NO MIN….93<br>DK……….98	[ ][ ]<br>NO MAX...93<br>DK……….98	YES . . .1<br>NO . . . .2 → **Q27e**<br>DK…….8 → **Q27e**	[ ][ ]	YES . . . .1<br>NO 2	YES . . . .1<br>NO 2	YES . . . .1<br>NO 2
(4) Male condoms	[ ][ ]<br>NO MIN….93<br>DK……….98	[ ][ ]<br>NO MAX...93<br>DK……….98	YES . . .1<br>NO . . . .2 → **Q27e**<br>DK…….8 → **Q27e**	[ ][ ]	YES . . . .1<br>NO 2	YES . . . .1<br>NO 2	YES . . . .1<br>NO 2
(5) Female condoms	[ ][ ]<br>NO MIN….93<br>DK……….98	[ ][ ]<br>NO MAX...93<br>DK……….98	YES . . .1<br>NO . . . .2 → **Q27e**<br>DK…….8 → **Q27e**	[ ][ ]	YES . . . .1<br>NO 2	YES . . . .1<br>NO 2	YES . . . .1<br>NO 2
(6) Spermicide (foam, foaming tablets, gel)	[ ][ ]<br>NO MIN….93<br>DK……….98	[ ][ ]<br>NO MAX...93<br>DK……….98	YES . . .1<br>NO . . . .2 → **Q27e**<br>DK…….8 → **Q27e**	[ ][ ]	YES . . . .1<br>NO 2	YES . . . .1<br>NO 2	YES . . . .1<br>NO 2
(7) Injectables (e.g. Depo Provera/ DMPA)	[ ][ ]<br>NO MIN….93<br>DK……….98	[ ][ ]<br>NO MAX...93<br>DK……….98	YES . . .1<br>NO . . . .2 → **Q27e**<br>DK…….8 → **Q27e**	[ ][ ]	YES . . . .1<br>NO 2	YES . . . .1<br>NO 2	YES . . . .1<br>NO 2
(8) Implant (e.g. Implanon or Jadelle)	[ ][ ]<br>NO MIN….93<br>DK……….98	[ ][ ]<br>NO MAX...93<br>DK……….98	YES . . .1<br>NO . . . .2 → **Q27e**<br>DK…….8 → **Q27e**	[ ][ ]	YES . . . .1<br>NO 2	YES . . . .1<br>NO 2	YES . . . .1<br>NO 2

Q28.	Is this pharmacy linked with any organization that provides family planning methods and materials at a discounted rate or for free (such as PPFN or SFH)?	Yes………………………………..1<br>No……………………………….2<br>Don't know………………………8	 **Q30**<br>**Q30**
Q29a.	What is the name of the organization?	Q29b What year did this facility begin to associate with each organization named?	
	1.	Year ……………... . . . . [ ][ ][ ][ ]<br>Don't know 9998	
	2.	Year ……………... . . . . [ ][ ][ ][ ]<br>Don't know 9998	
	3.	Year ……………... . . . . [ ][ ][ ][ ]<br>Don't know 9998	
	4.	Year ……………... . . . . [ ][ ][ ][ ]<br>Don't know 9998	
Q30.	Organizations like SFH and PPFN sometimes distribute products at a lower price to pharmacies to sell. These are called socially marketed products.<br>Do you have socially marketed contraceptive products in stock?	Yes . . . . 1<br>No . . . . 2<br>Don't know 8	 **Q32**<br>**Q32**
Q31.	What are all the socially marketed family planning products that you have in stock? **LIST SPECIFIC FAMILY PLANNING BRAND NAMES.**<br><br>**(CODE WILL BE PROVIDED AT THE OFFICE)**	________________________[__|__|__]<br>________________________[__|__|__]<br>________________________[__|__|__]	
**STORAGE & STOCK:** Now I would like to see the place where contraceptive methods are stored. We are just trying to get an idea of how we can help outlets improve their stocking and storing methods. Remember that my findings will be just used for research purposes and will be kept strictly confidential.			
Q32.	OBSERVE WHETHER ALL THE CONTRACEPTIVE METHODS ARE PROTECTED FROM WATER OR DAMPNESS	YES . . . . 1<br>NO . . . . 2<br>CANNOT OBSERVE STORAGE AREA………………..……………..3	 <br><br>**Q38**
Q33.	OBSERVE WHETHER ALL THE CONTRACEPTIVE METHODS ARE OFF THE FLOOR	YES . . . . 1<br>NO 2	
Q34.	OBSERVE WHETHER THE CEILING ABOVE THE CONTRACEPTIVE METHODS IS INTACT AND NOT LEAKING	YES . . . . 1<br>NO 2	
Q35.	OBSERVE WHETHER ALL THE CONTRACEPTIVE METHODS ARE PROTECTED FROM THE SUN.	YES . . . . 1<br>NO 2	
Q36.	OBSERVE WHETHER THE ROOM IS CLEAN OF EVIDENCE OF RODENTS (BATS, RATS) OR PESTS (ROACHES, ETC).	YES . . . . 1<br>NO 2	
Q37.	OBSERVE WHETHER THE INJECTABLES ARE STORED UPRIGHT.	YES . . . . 1<br>NO . . . . 2<br>NOT APPLICABLE/DON'T PROVIDE INJECTABLES……….7	
Q38.	Does the pharmacy separate damaged and/or expired family planning methods from the usable products, and remove them from the inventory?<br><br>IF YES, ASK TO SEE EVIDENCE OF EACH OF THE INDICATED PRACTICES AND ALL THAT WERE OBSERVED. ALSO ASK FOR THE TALLY CARD TO CHECK FOR RECORDED BALANCE.	YES, DAMAGED/EXPIRED ITEM REMOVED FROM INVENTORY . . . …………………. …... . . . . 1<br><br>REMOVED FROM SHELVES AND NO EXPIRED ITEMS PRESENT……………………………..….. ... . 2<br><br>EXPIRED ITEMS OBSERVED . . . . . . ……. .. 3<br><br>REPORTED YES BUT CANNOT OBSERVE….4<br><br>NO 5	

Q39.	OBSERVE WHETHER THERE IS A FUNCTIONAL REFRIGERATOR IN THE SHOP FOR STORING MEDICINES	YES, OBSERVED REFRIGERATOR AND FUNCTIONAL……………………..………………......1<br><br>YES, OBSERVED REFRIGERATOR BUT NOT FUNCTIONAL OR NOT USED FOR STORING MEDICINES…... ………………………….……..……….2<br><br>YES, BUT REFRIGERATOR NOT OBSERVED……..3<br><br>NO REFRIGERATOR PRESENT………………..…….5	

Q40.	RECORD THE TIME	Hour ………… ☐☐ Minutes …..☐☐
Thank you very much for taking the time to answer my questions. Once again, any information you have given will be kept confidential. Have a good day!		
COMMENTS:		
