# Supplementary material for: Contraceptive service provider imposed restrictions to contraceptive access in urban Nigeria
Source: BMC Health Serv Res. 2017 Apr 12;17:268. doi: 10.1186/s12913-017-2233-0 (PMC5389090; doi:10.1186/s12913-017-2233-0)
Supplement: Supplementary file 3 — PMS FINAL FROZEN. PMV Operators Survey. (PDF 252 kb) [file 12913_2017_2233_MOESM3_ESM.pdf]

MEASUREMENT, LEARNING & EVALUATION PROJECT
FOR THE URBAN REPRODUCTIVE HEALTH INITIATIVE

Measurement, Learning & Evaluation (MLE) Project
Chemists/Patent Medicine Stores (PMS) – Nigeria - 2011

CITY NAME & CODE____<br>(Abuja=1, Benin=2, Ibadan=3, Ilorin=4, Kaduna=5, Zaria=6)	[___]
LGA NAME & CODE____	[___|___|___]
LOCALITY NAME & CODE____	[___|___|___|___]
FACILITY NAME AND CODE____	[___|___|___|___|___]
PHYSICAL ADDRESS OF THIS STORE___________________________________	

LOCATION OF CHEMIST/PMS
GPS Reading

Altitude ………………………………….	[ ][ ][ ][ ]		
Latitude	N	[ ][ ]	[ ][ ][ ][ ][ ]
Longitude	E	[ ][ ][ ]	[ ][ ][ ][ ][ ]

INTERVIEWER VISITS

**VISIT No.**	**1**	**2**	**3**	**FINAL VISIT**
DATE	DAY/ MONTH/YEAR<br>[____/_____/_11_]	DAY/ MONTH/ YEAR<br>[____/_____/__11_]	DAY/ MONTH/ YEAR<br>[____/_____/__11_]	DAY [____|____]<br>MONTH [____|____]<br>YEAR [_2_|_0_|_1_|_1_]
INTERVIEWER'S NAME	_______________	_______________	_______________	____________________
INTERVIEWER CODE	[ ][ ][ ]	[ ][ ][ ]	[ ][ ][ ]	[ ][ ][ ]
RESULT*	[ ]	[ ]	[ ]	[ ]
**NEXT VISIT:**<br>**DATE**	[___/_____/11_]	[___/_____/11_]	[___/_____/11_]	TOTAL NO. OF VISITS<br>[ ]
**TIME**	[ ][ ] [ ][ ]<br>H H M M	[ ][ ] [ ][ ]<br>H H M M	[ ][ ] [ ][ ]<br>H H M M	

***RESULT CODES:**
1. COMPLETED
2. PHARMACY MOVED OR IS DESTROYED
3. RESPONDENT NOT AVAILABLE
4. RESPONDENT REFUSED
5. PARTLY COMPLETED
6. POSTPONED
7. OTHER ___________________________________
(SPECIFY)

	ENGLISH	HAUSA	YORUBA	IGBO	PIDGIN	OTHER(SPECIFY)	TRANSLATOR USED?	
							YES	NO
LANGUAGE OF INTERVIEW	1	2	3	4	5	6___________	1	2
NATIVE LANGUAGE OF RESPONDENT	1	2	3	4	5	6___________		

**FIND THE PERSON WHO IS THE OWNER OF THE SHOP. IF HE/SHE IS NOT PRESENT, ASK TO SEE THE PERSON MOST KNOWLEDGEABLE ABOUT THE INVENTORY.**

POSITION OF RESPONDENT<br>1 = OWNER<br>2 = EMPLOYEE<br>3= TRAINEE/APPRENTICE<br>6 = OTHER: ____________________________<br>(SPECIFY)	SEX OF RESPONDENT<br>1= MALE<br>2= FEMALE

**SUPERVISOR**	**OFFICE EDITOR**	**KEYED BY**
NAME………………………………	NAME……………………………….	NAME…………………………………………..
CODE: [ ][ ][ ]	CODE: [ ][ ][ ]	CODE: [ ][ ][ ]
DATE [__ / ___/ 11_]<br>DD MM YY	DATE [__ / ___/ 11_]<br>DD MM YY	DATE [__/ __/ 11_]<br>DD MM YY

Q1.	RECORD THE TIME<br>**(IN 24 HOUR FORMAT)**	Hour ……….[ ][ ] Minutes ….…..[ ][ ]	
**GENERAL INFORMATION**			
**Source**	**Questions**	**Coding**	**Skip**
Q2.	What is the highest level of school you attended: Quranic only, primary, junior secondary, senior secondary, or higher?	QURANIC ONLY……………………..0 →<br>PRIMARY……………………………1<br>JUNIOR SECONDARY (JSS)………..2<br>SENIOR SECONDARY (SSS)……….3<br>HIGHER……………………………..4<br>DID NOT ATTEND SCHOOL…………5 →	**Q4**<br><br><br><br><br>**Q4**
Q3.	What is the highest (class/form/year) you completed at that level?	CLASS/FORM/YEAR……………….[__|__]	
Q4.	Do you have any professional medical qualification?	YES………………………………..1<br>NO…………………………………2 →	**Q6**
Q5.	What is your medical qualification?	PHYSICIAN……………………………………01<br>REGISTERED PHARMACIST………………..02<br>PHARMACY TECHNICIAN……………………03<br>NURSE…………………………………………..04<br>MIDWIFE………………………………………..05<br>NURSE/ MIDWIFE……………………………..06<br>CHEW…………………………………………..07<br>CHO……………………………………………..08<br>OTHER _____________________________96	
Q6.	In this shop, how many regular, permanent staff (workers) work here?	[ ][ ]	
Q7.	Do you have an official training program for PMV trainees?	YES………………………………..1<br>NO…………………………………2 →	**Q9**
Q8.	How many trainees are you currently training?	[ ][ ]	

Q9.	In what year did this shop open?<br><br>PROBE, IF RESPONDANT SAYS DON'T KNOW: THIS IS VERY IMPORTANT.<br>Can you tell me how old this shop is? For example, would you say it is about 7 years old? 10 years old? (etc.)	FILL IN EITHER YEAR OPENED **OR** YEARS OLD:<br>YEAR OPENED…..[ ][ ][ ][ ]<br>DON'T KNOW……9998<br>**OR**<br>YEARS OLD . . . . . .[ ][ ]	
Q10.	On average, how many hours per day is the shop open?	HOURS PER DAY . . . .[ ][ ]	
Q11.	On average, how many days per week is the shop open?	DAYS PER WEEK . . . .[ ]	

Q12.	Do you belong to any trade-related association(s)?	YES……………………………………….1<br>NO……………………………………2 →<br>IN PROCESS………………………3 →<br>DON'T KNOW………………………8 →	 **Q14**<br>**Q14**<br>**Q14**
Q13.	If yes, which association(s):	1.________________________________<br>2.________________________________<br>3.________________________________	
Q14.	Do you belong to any health-related association(s)?	YES……………………………………….1<br>NO…………………………………2 →<br>IN PROCESS……………………3 →<br>DON'T KNOW……………………8 →	 **Q16a**<br>**Q16a**<br>**Q16a**
Q15.	If yes, which association(s):	1.________________________________<br>2.________________________________<br>3.________________________________	
Q16a.	Is there a stock register for family planning methods received, the amount disbursed, and the amount present today (stock balance) is recorded?	YES, OBSERVED . . . . 1<br>YES, REPORTED, NOT SEEN . . . 2<br>NO 3	
Q16b.	How often do you update or reconcile your inventory/stock records?	EVERY [ ][ ] DAY(S)<br><br>THE DAY ITEMS ARE<br>RECEIVED OR DISBURSED . ……. . . 95<br>NEVER . . . . ….. . .. . . 97<br>OTHER________________________96<br>(SPECIFY)	
Q16c	Is the stock maintenance system computerized?	YES . . . . 1<br>NO 2	
Q16d.	CIRCLE THE RESPONSE THAT BEST DESCRIBES THE SYSTEM.	STOCK RECORDS UPDATED ON THE DAY ITEM RECEIVED/DISBURSED…………………………….1<br><br>STOCK RECORDS NOT ALWAYS UPDATED WHEN ITEM DISBURSED, BUT RECORD OF ITEMS RECEIVED /DISTRIBUTED OBSERVED……..…..2<br><br>NO RECORDS OBSERVED……………………..……3<br><br>RECORDS NOT UP TO DATE…………………..…4<br><br>OTHER________________________________6<br>(SPECIFY)	
Q17.	Have you received any training on family planning?	YES………………………………..1<br>NO…………………………………2 →<br>DON'T KNOW……………………8 →	 **Q20**<br>**Q20**
Q18.	When was the last family planning training that you attended?	DAYS AGO………1<br>WEEKS AGO……2 [ ][ ]<br>MONTHS AGO….3<br>YEARS AGO…….4	
Q19.	What were the issues covered in the last training?<br><br>**CIRCLE ALL MENTIONED.**	CONTRACEPTIVE TECHNOLOGY UPDATE…A<br>EXCLUSIVE BREASTFEEDING COUNSELING/LAM…………………………….…B<br>NATURAL FP (STANDARD DAYS, CYCLE BEADS, ETC.)…………………………………….C<br>FP COUNSELING SKILLS………………………D<br>PILLS……………………………………………..E<br>CONDOMS………………………………………..F<br>SPERMICIDE……………………………………..G<br>EMERGENCY CONTRACEPTIVE……….……H<br>IUD…………………………………….…………I<br>INJECTABLES……………………………………J<br>DIAPHRAGM……………………………………..K<br>OTHERS _____________________________X<br>(SPECIFY)	

Q20.	Would you be willing to attend any future training on family planning/birth spacing or other reproductive health needs?	YES……………………………………1<br>NO . . . . .2<br>DON'T KNOW 8	

Q21.	Do you talk about family planning/birth spacing to your customers/clients?	YES……………………………………1 →<br>NO . . . . .2<br>DON'T KNOW 8	**Q23**
Q22.	Would you be willing to talk about family planning/birth spacing to customers/clients?	YES……………………………………1<br>NO . . . . .2<br>DON'T KNOW 8	
Q23.	Do you refer clients/customers to other stores or facilities for family planning/birth spacing methods?	YES………………………………… .1<br>NO . . . . .2<br>DON'T KNOW 8	
Q24.	Do you provide materials on family planning/birth spacing to your customers/clients?	YES……………………………………1 →<br>NO . . . . .2<br>DON'T KNOW 8	**Q26**
Q25.	Would you be willing to provide materials on family planning/birth spacing to customers/clients?	YES……………………………………1<br>NO . . . . .2<br>DON'T KNOW 8	
Q26.	OBSERVE WHETHER THERE ARE ANY FAMILY PLANNING PROMOTIONAL MATERIALS ON DISPLAY (EG, POSTERS, BROCHURES, DANGLERS, CALENDARS, ETC.)	DISPLAYED………………………..1<br>NOT DISPLAYED………………….2	
Q27.	Would you be willing to display (additional) information/educational materials on family planning/birth spacing at this shop?	YES……………………………………1<br>NO . . . . .2<br>DON'T KNOW 8	
Q28.	Do you provide family planning/birth spacing methods at this shop?	YES………………………………… .1 →<br>NO . . . . .2<br>DON'T KNOW 8	**Q32a**
Q29.	Would you be willing to sell family planning/birth spacing methods at this shop?	YES……………………………………1<br>NO . . . . .2 →<br>DON'T KNOW . . . . .8 →	 **Q31**<br>**Q31**
Q30.	Which methods would you be willing to sell?<br><br>CIRCLE ALL MENTIONED.	COMBINED PILL………….. ………………..A<br>PROGESTIN-ONLY PILL.............................B<br>PILL (UNSPECIFIED)..................................C<br>MALE CONDOM ... . ………………. D<br>FEMALE CONDOM . .. . . …. . . …………….E<br>IUD……..... . . . . …………… F<br>SPERMICIDE……………….....……………… G<br>DIAPHRAGM . . . . ……………….H<br>INJECTABLES.. . . . . ……………I<br>IMPLANT . . . . ……………J<br>EMERGENCY CONTRACEPTION . . . . .K<br>OTHER ____________________________X<br>(SPECIFY)	
Q31.	What would help influence you to decide to provide family planning information and methods?<br><br>CIRCLE ALL MENTIONED.	FREE TRAINING…………………………….A<br>FREE PRODUCTS…………………………B<br>REDUCED PRICE OF PRODUCTS………..C<br>FREE PROMOTIONAL MATERIALS………D<br>OTHER ____________________________X<br>(SPECIFY)<br>NOTHING………………………………………Y<br>} →	**END**

ASK IF THE FOLLOWING CONTRACEPTIVES ARE AVAILABLE. FOR EACH ITEM, CIRCLE THE APPROPRIATE CODE.

FOR ALL ITEMS THAT ARE OBSERVED, ASK IF THERE HAS BEEN ANY STOCK-OUT (BRAND NOT AVAILABLE FOR AT LEAST 24 HOURS) DURING THE LAST 12 MONTHS AND LAST 30 DAYS.

CONTRA-CEPTIVE<br><br>(ASK FOR MOST POPULAR BRANDS)	Q32a. Does this pharmacy usually sell the following FP methods?	Q32b. What brands do you <u>usually</u> stock? **LIST ALL BRAND NAMES USUALLY STOCKED, EVEN IF CURRENTLY OUT OF STOCK. IF THERE ARE MORE THAN 3 BRANDS, LIST THE 3 MOST POPULAR BRANDS** (CODING WILL BE DONE IN THE OFFICE).	Q32c. What is the retail price (in Naira) for ______ [PRODUCT/ BRAND]?	Q32d. What is the average retail sales volume in a month?	Q32e. Is ________ [PRODUCT/ BRAND] currently available?	Q32f. Has ______ [PRODUCT/ BRAND] been stocked out in this store for at least 24 hours in the last **ONE** year? **IF NO OR DON'T KNOW, SKIP TO NEXT BRAND.**	Q32g. In the past one year, for how many total days were you stocked out of ________ [PRODUCT/ BRAND] (all stock-outs combined)?	Q32h. Has ____ [PRODUCT/ BRAND] been stocked out for at least 24 hours in the last **ONE** month (30 days)? **IF NO OR DON'T KNOW, SKIP TO NEXT BRAND/ METHOD.**	Q32i. In the last 30 days, for how many total days were you stocked out of _____ [PRODUCT/ BRAND] (all stock-outs combined)?
(1) Combin-ation oral contra-ceptives (estrogen and pro-gestin)	YES..1<br>NO…2→**(2)**	____________[__|__|__]<br>BRAND (1)<br><br>____________[__|__|__]<br>BRAND (2)<br><br>____________[__|__|__]<br>BRAND (3)	RETAIL PRICE PER CYCLE:<br><br>[__|__|__|__|__]<br>BRAND (1)<br><br>[__|__|__|__|__]<br>BRAND (2)<br><br>[__|__|__|__|__]<br>BRAND (3)	SALES VOLUME (CYCLES):<br><br>[__|__|__|__|__]<br>BRAND (1)<br><br>[__|__|__|__|__]<br>BRAND (2)<br><br>[__|__|__|__|__]<br>BRAND (3)	YES . .1<br>NO …..2<br><br>BRAND (1)<br><br>BRAND (2)<br><br>BRAND (3)	YES….1<br>NO …..2<br>DK…….8<br><br>BRAND (1)<br><br>BRAND (2)<br><br>BRAND (3)	RECORD DAYS:<br><br>BRAND (1)<br>Don't know…998<br><br>BRAND (2)<br>Don't know…998<br><br>BRAND (3)<br>Don't know…998	YES….1<br>NO …..2<br>DK…….8<br><br>BRAND (1)<br><br>BRAND (2)<br><br>BRAND (3)	RECORD DAYS:<br><br>BRAND (1)<br>Don't know…98<br><br>BRAND (2)<br>Don't know…98<br><br>BRAND (3)<br>Don't know…98
(2) Progestin-only oral contra-ceptives	YES..1<br>NO…2→**(3)**	____________[__|__|__]<br>BRAND (1)<br><br>____________[__|__|__]<br>BRAND (2)<br><br>____________[__|__|__]<br>BRAND (3)	RETAIL PRICE PER CYCLE:<br><br>[__|__|__|__|__]<br>BRAND (1)<br><br>[__|__|__|__|__]<br>BRAND (2)<br><br>[__|__|__|__|__]<br>BRAND (3)	SALES VOLUME (CYCLES):<br><br>[__|__|__|__|__]<br>BRAND (1)<br><br>[__|__|__|__|__]<br>BRAND (2)<br><br>[__|__|__|__|__]<br>BRAND (3)	YES . .1<br>NO …..2<br><br>BRAND (1)<br><br>BRAND (2)<br><br>BRAND (3)	YES….1<br>NO …..2<br>DK…….8<br><br>BRAND (1)<br><br>BRAND (2)<br><br>BRAND (3)	RECORD DAYS:<br><br>BRAND (1)<br>Don't know…998<br><br>BRAND (2)<br>Don't know…998<br><br>BRAND (3)<br>Don't know…998	YES….1<br>NO …..2<br>DK…….8<br><br>BRAND (1)<br><br>BRAND (2)<br><br>BRAND (3)	RECORD DAYS:<br><br>BRAND (1)<br>Don't know…98<br><br>BRAND (2)<br>Don't know…98<br><br>BRAND (3)<br>Don't know…98

CONTRA-CEPTIVE<br><br>(ASK FOR MOST POPULAR BRANDS)	Q32a. Does this pharmacy usually sell the following FP methods?	Q32b. What brands do you usually stock?<br>**LIST ALL BRAND NAMES USUALLY STOCKED, EVEN IF CURRENTLY OUT OF STOCK. IF THERE ARE MORE THAN 3 BRANDS, LIST THE 3 MOST POPULAR BRANDS** (CODING WILL BE DONE IN THE OFFICE).	Q32c. What is the retail price (in Naira) for ______ [PRODUCT/ BRAND]?	Q32d. What is the average retail sales volume in a month?	Q32e. Is ________ [PRODUCT/ BRAND] currently available?	Q32f. Has ______ [PRODUCT/ BRAND] been stocked out in this store for at least 24 hours in the last **ONE** year?<br>**IF NO OR DON'T KNOW, SKIP TO NEXT BRAND.**	Q32g. In the past one year, for how many total days were you stocked out of ________ [PRODUCT/ BRAND] (all stock-outs combined)?	Q32h. Has ____ [PRODUCT/ BRAND] been stocked out for at least 24 hours in the last **ONE** month (30 days)?<br>**IF NO OR DON'T KNOW, SKIP TO NEXT BRAND/ METHOD.**	Q32i. In the last 30 days, for how many total days were you stocked out of _____ [PRODUCT/ BRAND] (all stock-outs combined)?
(3) Emergency contraceptives	YES..1<br>NO…2→**(4)**	____________[__|__|__]<br>BRAND (1)<br><br>____________[__|__|__]<br>BRAND (2)<br><br>____________[__|__|__]<br>BRAND (3)	RETAIL PRICE PER PACK:<br><br>[__|__|__|__|__]<br>BRAND (1)<br><br>[__|__|__|__|__]<br>BRAND (2)<br><br>[__|__|__|__|__]<br>BRAND (3)	SALES VOLUME (PACKS):<br><br>[__|__|__|__|__]<br>BRAND (1)<br><br>[__|__|__|__|__]<br>BRAND (2)<br><br>[__|__|__|__|__]<br>BRAND (3)	YES . .1<br>NO …..2<br><br>☐<br>BRAND (1)<br><br>☐<br>BRAND (2)<br><br>☐<br>BRAND (3)	YES….1<br>NO …..2<br>DK…….8<br><br>☐<br>BRAND (1)<br><br>☐<br>BRAND (2)<br><br>☐<br>BRAND (3)	RECORD DAYS:<br><br>☐☐☐<br>BRAND (1)<br>Don't know…998<br><br>☐☐☐<br>BRAND (2)<br>Don't know…998<br><br>☐☐☐<br>BRAND (3)<br>Don't know…998	YES….1<br>NO …..2<br>DK…….8<br><br>☐<br>BRAND (1)<br><br>☐<br>BRAND (2)<br><br>☐<br>BRAND (3)	RECORD DAYS:<br><br>☐☐<br>BRAND (1)<br>Don't know…98<br><br>☐☐<br>BRAND (2)<br>Don't know…98<br><br>☐☐<br>BRAND (3)<br>Don't know…98
(4) Male condoms	YES..1<br>NO…2→**(5)**	____________[__|__|__]<br>BRAND (1)<br><br>____________[__|__|__]<br>BRAND (2)<br><br>____________[__|__|__]<br>BRAND (3)	RETAIL PRICE PER PIECE:<br><br>[__|__|__|__|__]<br>BRAND (1)<br><br>[__|__|__|__|__]<br>BRAND (2)<br><br>[__|__|__|__|__]<br>BRAND (3)	SALES VOLUME (PIECES):<br><br>[__|__|__|__|__]<br>BRAND (1)<br><br>[__|__|__|__|__]<br>BRAND (2)<br><br>[__|__|__|__|__]<br>BRAND (3)	YES . .1<br>NO …..2<br><br>☐<br>BRAND (1)<br><br>☐<br>BRAND (2)<br><br>☐<br>BRAND (3)	YES….1<br>NO …..2<br>DK…….8<br><br>☐<br>BRAND (1)<br><br>☐<br>BRAND (2)<br><br>☐<br>BRAND (3)	RECORD DAYS:<br><br>☐☐☐<br>BRAND (1)<br>Don't know…998<br><br>☐☐☐<br>BRAND (2)<br>Don't know…998<br><br>☐☐☐<br>BRAND (3)<br>Don't know…998	YES….1<br>NO …..2<br>DK…….8<br><br>☐<br>BRAND (1)<br><br>☐<br>BRAND (2)<br><br>☐<br>BRAND (3)	RECORD DAYS:<br><br>☐☐<br>BRAND (1)<br>Don't know…98<br><br>☐☐<br>BRAND (2)<br>Don't know…98<br><br>☐☐<br>BRAND (3)<br>Don't know…98

CONTRA-CEPTIVE<br><br>(ASK FOR MOST POPULAR BRANDS)	Q32a. Does this pharmacy usually sell the following FP methods?	Q32b. What brands do you usually stock? **LIST ALL BRAND NAMES USUALLY STOCKED, EVEN IF CURRENTLY OUT OF STOCK. IF THERE ARE MORE THAN 3 BRANDS, LIST THE 3 MOST POPULAR BRANDS** (CODING WILL BE DONE IN THE OFFICE).	Q32c. What is the retail price (in Naira) for ______ [PRODUCT/ BRAND]?	Q32d. What is the average retail sales volume in a month?	Q32e. Is ________ [PRODUCT/ BRAND] currently available?	Q32f. Has ______ [PRODUCT/ BRAND] been stocked out in this store for at least 24 hours in the last **ONE** year? **IF NO OR DON'T KNOW, SKIP TO NEXT BRAND.**	Q32g. In the past one year, for how many total days were you stocked out of ________ [PRODUCT/ BRAND] (all stock-outs combined)?	Q32h. Has ____ [PRODUCT/ BRAND] been stocked out for at least 24 hours in the last **ONE** month (30 days)? **IF NO OR DON'T KNOW, SKIP TO NEXT BRAND/ METHOD.**	Q32i. In the last 30 days, for how many total days were you stocked out of _____ [PRODUCT/ BRAND] (all stock-outs combined)?
(5) Female condoms	YES..1<br>NO…2→**(6)**	____________[__|__|__]<br>BRAND (1)<br><br>____________[__|__|__]<br>BRAND (2)<br><br>____________[__|__|__]<br>BRAND (3)	RETAIL PRICE PER PIECE:<br><br>[__|__|__|__|__]<br>BRAND (1)<br><br>[__|__|__|__|__]<br>BRAND (2)<br><br>[__|__|__|__|__]<br>BRAND (3)	SALES VOLUME (PIECES):<br><br>[__|__|__|__|__]<br>BRAND (1)<br><br>[__|__|__|__|__]<br>BRAND (2)<br><br>[__|__|__|__|__]<br>BRAND (3)	YES . .1<br>NO …..2<br><br>☐ BRAND (1)<br><br>☐ BRAND (2)<br><br>☐ BRAND (3)	YES….1<br>NO …..2<br>DK…….8<br><br>☐ BRAND (1)<br><br>☐ BRAND (2)<br><br>☐ BRAND (3)	RECORD DAYS:<br><br>☐☐☐ BRAND (1)<br>Don't know…998<br><br>☐☐☐ BRAND (2)<br>Don't know…998<br><br>☐☐☐ BRAND (3)<br>Don't know…998	YES….1<br>NO …..2<br>DK…….8<br><br>☐ BRAND (1)<br><br>☐ BRAND (2)<br><br>☐ BRAND (3)	RECORD DAYS:<br><br>☐☐ BRAND (1)<br>Don't know…98<br><br>☐☐ BRAND (2)<br>Don't know…98<br><br>☐☐ BRAND (3)<br>Don't know…98
(6) Spermicide (foam, foaming tablets, gel)	YES..1<br>NO…2→**(7)**	____________[__|__|__]<br>BRAND (1)<br><br>____________[__|__|__]<br>BRAND (2)<br><br>____________[__|__|__]<br>BRAND (3)	RETAIL PRICE PER UNIT:<br><br>[__|__|__|__|__]<br>BRAND (1)<br><br>[__|__|__|__|__]<br>BRAND (2)<br><br>[__|__|__|__|__]<br>BRAND (3)	SALES VOLUME (UNITS):<br><br>[__|__|__|__|__]<br>BRAND (1)<br><br>[__|__|__|__|__]<br>BRAND (2)<br><br>[__|__|__|__|__]<br>BRAND (3)	YES . .1<br>NO …..2<br><br>☐ BRAND (1)<br><br>☐ BRAND (2)<br><br>☐ BRAND (3)	YES….1<br>NO …..2<br>DK…….8<br><br>☐ BRAND (1)<br><br>☐ BRAND (2)<br><br>☐ BRAND (3)	RECORD DAYS:<br><br>☐☐☐ BRAND (1)<br>Don't know…998<br><br>☐☐☐ BRAND (2)<br>Don't know…998<br><br>☐☐☐ BRAND (3)<br>Don't know…998	YES….1<br>NO …..2<br>DK…….8<br><br>☐ BRAND (1)<br><br>☐ BRAND (2)<br><br>☐ BRAND (3)	RECORD DAYS:<br><br>☐☐ BRAND (1)<br>Don't know…98<br><br>☐☐ BRAND (2)<br>Don't know…98<br><br>☐☐ BRAND (3)<br>Don't know…98

CONTRA-CEPTIVE<br><br>(ASK FOR MOST POPULAR BRANDS)	Q32a. Does this pharmacy usually sell the following FP methods?	Q32b. What brands do you usually stock?<br>**LIST ALL BRAND NAMES USUALLY STOCKED, EVEN IF CURRENTLY OUT OF STOCK. IF THERE ARE MORE THAN 3 BRANDS, LIST THE 3 MOST POPULAR BRANDS** (CODING WILL BE DONE IN THE OFFICE).	Q32c. What is the retail price (in Naira) for ______ [PRODUCT/ BRAND]?	Q32d. What is the average retail sales volume in a month?	Q32e. Is _________ [PRODUCT/ BRAND] currently available?	Q32f. Has ______ [PRODUCT/ BRAND] been stocked out in this store for at least 24 hours in the last **ONE** year?<br>**IF NO OR DON'T KNOW, SKIP TO NEXT BRAND.**	Q32g. In the past one year, for how many total days were you stocked out of ________ [PRODUCT/ BRAND] (all stock-outs combined)?	Q32h. Has ____ [PRODUCT/ BRAND] been stocked out for at least 24 hours in the last **ONE** month (30 days)?<br>**IF NO OR DON'T KNOW, SKIP TO NEXT BRAND/ METHOD.**	Q32i. In the last 30 days, for how many total days were you stocked out of _____ [PRODUCT/ BRAND] (all stock-outs combined)?
(7) Injectables (Depo, Noristerat)	YES..1<br>NO…2→**(8)**	______________[__|__|__]<br>BRAND (1)<br><br>______________[__|__|__]<br>BRAND (2)<br><br>______________[__|__|__]<br>BRAND (3)	RETAIL PRICE PER INJECTABLE:<br><br>[__|__|__|__]<br>BRAND (1)<br><br>[__|__|__|__]<br>BRAND (2)<br><br>[__|__|__|__]<br>BRAND (3)	SALES VOLUME (INJECTS)<br><br>[__|__|__|__]<br>BRAND (1)<br><br>[__|__|__|__]<br>BRAND (2)<br><br>[__|__|__|__]<br>BRAND (3)	YES . .1<br>NO …..2<br><br>☐<br>BRAND (1)<br><br>☐<br>BRAND (2)<br><br>☐<br>BRAND (3)	YES….1<br>NO …..2<br>DK…….8<br><br>☐<br>BRAND (1)<br><br>☐<br>BRAND (2)<br><br>☐<br>BRAND (3)	RECORD DAYS:<br><br>☐☐☐<br>BRAND (1)<br>Don't know…998<br><br>☐☐☐<br>BRAND (2)<br>Don't know…998<br><br>☐☐☐<br>BRAND (3)<br>Don't know…998	YES….1<br>NO …..2<br>DK…….8<br><br>☐<br>BRAND (1)<br><br>☐<br>BRAND (2)<br><br>☐<br>BRAND (3)	RECORD DAYS:<br><br>☐☐<br>BRAND (1)<br>Don't know…98<br><br>☐☐<br>BRAND (2)<br>Don't know…98<br><br>☐☐<br>BRAND (3)<br>Don't know…98
(8) Implant (e.g. Implanon or Jadelle)	YES..1<br>NO…2→ **(Q33a)**	______________[__|__|__]<br>BRAND (1)<br><br>______________[__|__|__]<br>BRAND (2)<br><br>______________[__|__|__]<br>BRAND (3)	RETAIL PRICE PER IMPLANT:<br><br>[__|__|__|__]<br>BRAND (1)<br><br>[__|__|__|__]<br>BRAND (2)<br><br>[__|__|__|__]<br>BRAND (3)	SALES VOLUME (IMPLANTS):<br><br>[__|__|__|__]<br>BRAND (1)<br><br>[__|__|__|__]<br>BRAND (2)<br><br>[__|__|__|__]<br>BRAND (3)	YES . .1<br>NO …..2<br><br>☐<br>BRAND (1)<br><br>☐<br>BRAND (2)<br><br>☐<br>BRAND (3)	YES….1<br>NO …..2<br>DK…….8<br><br>☐<br>BRAND (1)<br><br>☐<br>BRAND (2)<br><br>☐<br>BRAND (3)	RECORD DAYS:<br><br>☐☐☐<br>BRAND (1)<br>Don't know…998<br><br>☐☐☐<br>BRAND (2)<br>Don't know…998<br><br>☐☐☐<br>BRAND (3)<br>Don't know…998	YES….1<br>NO …..2<br>DK…….8<br><br>☐<br>BRAND (1)<br><br>☐<br>BRAND (2)<br><br>☐<br>BRAND (3)	RECORD DAYS:<br><br>☐☐<br>BRAND (1)<br>Don't know…98<br><br>☐☐<br>BRAND (2)<br>Don't know…98<br><br>☐☐<br>BRAND (3)<br>Don't know…98

Now I would like to ask you about your specific stocks of different family planning methods/products.<br>**ONLY ASK ABOUT THOSE METHODS THAT ARE AVAILABLE FROM Q32a.**		
CONTRACEPTIVE	Q33a. Where does your stock of CONTRACEPTIVE (most popular brands) come from? CHOOSE ALL.	Q33b. On average, how long does it take to receive your supplies after you have placed an order? READ LIST.
(01) Combination oral contraceptives (estrogen and progestin)	Government……………………………… A<br>Intl NGO ………………………………… B<br>Local NGO……………………………...…C<br>Pharmacy wholesaler/distributor……...D<br>Other_____________________________X<br>(Specify)<br>Don't know……………………………...…Z	One week or less…....1<br>Between 2-4 weeks….2<br>Between 5-8 weeks….3<br>More than 8 weeks…..4<br>Other ____________6<br>(Specify)<br>Don't know . ………….8
(02) Progestin-only oral contraceptives	Government……………………………… A<br>Intl NGO ………………………………… B<br>Local NGO……………………………...…C<br>Pharmacy wholesaler/distributor……...D<br>Other_____________________________X<br>(Specify)<br>Don't know……………………………...…Z	One week or less…....1<br>Between 2-4 weeks….2<br>Between 5-8 weeks….3<br>More than 8 weeks…..4<br>Other ____________6<br>(Specify)<br>Don't know . ………….8
(03) Emergency contraceptives	Government……………………………… A<br>Intl NGO ………………………………… B<br>Local NGO……………………………...…C<br>Pharmacy wholesaler/distributor……...D<br>Other_____________________________X<br>(Specify)<br>Don't know……………………………...…Z	One week or less…....1<br>Between 2-4 weeks….2<br>Between 5-8 weeks….3<br>More than 8 weeks…..4<br>Other ____________6<br>(Specify)<br>Don't know . ………….8
(04) Male condoms	Government……………………………… A<br>Intl NGO ………………………………… B<br>Local NGO……………………………...…C<br>Pharmacy wholesaler/distributor……...D<br>Other_____________________________X<br>(Specify)<br>Don't know……………………………...…Z	One week or less…....1<br>Between 2-4 weeks….2<br>Between 5-8 weeks….3<br>More than 8 weeks…..4<br>Other ____________6<br>(Specify)<br>Don't know . ………….8
(05) Female condoms	Government……………………………… A<br>Intl NGO ………………………………… B<br>Local NGO……………………………...…C<br>Pharmacy wholesaler/distributor……...D<br>Other_____________________________X<br>(Specify)<br>Don't know……………………………...…Z	One week or less…....1<br>Between 2-4 weeks….2<br>Between 5-8 weeks….3<br>More than 8 weeks…..4<br>Other ____________6<br>(Specify)<br>Don't know . ………….8
(06) Spermicide	Government……………………………… A<br>Intl NGO ………………………………… B<br>Local NGO……………………………...…C<br>Pharmacy wholesaler/distributor……...D<br>Other_____________________________X<br>(Specify)<br>Don't know……………………………...…Z	One week or less…....1<br>Between 2-4 weeks….2<br>Between 5-8 weeks….3<br>More than 8 weeks…..4<br>Other ____________6<br>(Specify)<br>Don't know . ………….8
(07) Injectables (e.g., Depo Provera, Noristerat)	Government……………………………… A<br>Intl NGO ………………………………… B<br>Local NGO……………………………...…C<br>Pharmacy wholesaler/distributor……...D<br>Other_____________________________X<br>(Specify)<br>Don't know……………………………...…Z	One week or less…....1<br>Between 2-4 weeks….2<br>Between 5-8 weeks….3<br>More than 8 weeks…..4<br>Other ____________6<br>(Specify)<br>Don't know . ………….8
(08) Implant (Norplant)	Government……………………………… A<br>Intl NGO ………………………………… B<br>Local NGO……………………………...…C<br>Pharmacy wholesaler/distributor……...D<br>Other_____________________________X<br>(Specify)<br>Don't know……………………………...…Z	One week or less…....1<br>Between 2-4 weeks….2<br>Between 5-8 weeks….3<br>More than 8 weeks…..4<br>Other ____________6<br>(Specify)<br>Don't know . ………….8

Q34.	If there is a shortage of a specific CONTRACEPTIVE between routine orders, what is the <u>most common</u> procedure followed by this pharmacy?<br><br>- Submit special order to normal supplier<br><br>- Pharmacy purchases from private market<br><br>- Clients must purchase from another outlet<br><br>- Facility borrows from neighboring Pharmacy<br><br>- None of the above	SPECIAL ORDER . . . .1<br><br>PHARMACY PURCHASE . . . . 2<br><br>CLIENTS PURCHASE ELSEWHERE. ...3<br><br>PHARMACY BORROWS . . . . .4<br><br>NONE OF THE ABOVE 5	→ **Q36**<br><br>→ **Q36**<br><br>→ **Q36**<br><br><br><br>→ **Q36**
Q35.	When you borrow CONTRACEPTIVE supplies, from what outlet do you most often borrow?	NAME:<br>_______________________________________	
Q36.	From which type of outlet do you borrow CONTRACEPTIVE supplies?	Government……………………………...……………..1<br>Private (for-profit)…………………………….……….2<br>NGO (not-for profit)…..………………………….………3<br>Mission……………….…………………….…………4<br>Other ______________________________________6<br>(Specify)	

ASK ONLY ABOUT THE FAMILY PLANNING METHODS THE PHARMACY IS PROVIDING IN Q32a.
Now I would like to ask you specifically about the contraceptive methods that you provide.

CONTRACEPTIVE	Q37a. What is the minimum age that you would offer this METHOD?	Q37b. What is the maximum age that you would offer this METHOD?	Q37c. Is there a minimum number of children a person must have before you will offer METHOD?	Q37d. What is that minimum number of children?	Q37e. Do you require a partner's consent before you will provide METHOD?	Q37f. Would you offer METHOD to an unmarried person?	Q37g. Do you require a prescription for a client to receive this METHOD?
(1) Combination oral contraceptives (estrogen and progestin)	__ __<br>NO MIN….93<br>DK……….98	__ __<br>NO MAX...93<br>DK……….98	YES . . .1<br>NO . . . .2 → **Q37e**<br>DK…….8 → **Q37e**	__ __	YES . . . .1<br>NO 2	YES . . . .1<br>NO 2	YES . . . .1<br>NO 2
(2) Progestin-only oral contraceptives	__ __<br>NO MIN….93<br>DK……….98	__ __<br>NO MAX...93<br>DK……….98	YES . . .1<br>NO . . . .2 → **Q37e**<br>DK…….8 → **Q37e**	__ __	YES . . . .1<br>NO 2	YES . . . .1<br>NO 2	YES . . . .1<br>NO 2
(3) Emergency contraceptives	__ __<br>NO MIN….93<br>DK……….98	__ __<br>NO MAX...93<br>DK……….98	YES . . .1<br>NO . . . .2 → **Q37e**<br>DK…….8 → **Q37e**	__ __	YES . . . .1<br>NO 2	YES . . . .1<br>NO 2	YES . . . .1<br>NO 2
(4) Male condoms	__ __<br>NO MIN….93<br>DK……….98	__ __<br>NO MAX...93<br>DK……….98	YES . . .1<br>NO . . . .2 → **Q37e**<br>DK…….8 → **Q37e**	__ __	YES . . . .1<br>NO 2	YES . . . .1<br>NO 2	YES . . . .1<br>NO 2
(5) Female condoms	__ __<br>NO MIN….93<br>DK……….98	__ __<br>NO MAX...93<br>DK……….98	YES . . .1<br>NO . . . .2 → **Q37e**<br>DK…….8 → **Q37e**	__ __	YES . . . .1<br>NO 2	YES . . . .1<br>NO 2	YES . . . .1<br>NO 2
(6) Spermicide (foam, foaming tablets, gel)	__ __<br>NO MIN….93<br>DK……….98	__ __<br>NO MAX...93<br>DK……….98	YES . . .1<br>NO . . . .2 → **Q37e**<br>DK…….8 → **Q37e**	__ __	YES . . . .1<br>NO 2	YES . . . .1<br>NO 2	YES . . . .1<br>NO 2
(7) Injectables (e.g. Depo Provera/ DMPA)	__ __<br>NO MIN….93<br>DK……….98	__ __<br>NO MAX...93<br>DK……….98	YES . . .1<br>NO . . . .2 → **Q37e**<br>DK…….8 → **Q37e**	__ __	YES . . . .1<br>NO 2	YES . . . .1<br>NO 2	YES . . . .1<br>NO 2
(8) Implant (e.g. Implanon or Jadelle)	__ __<br>NO MIN….93<br>DK……….98	__ __<br>NO MAX...93<br>DK……….98	YES . . .1<br>NO . . . .2 → **Q37e**<br>DK…….8 → **Q37e**	__ __	YES . . . .1<br>NO 2	YES . . . .1<br>NO 2	YES . . . .1<br>NO 2

Q38.	Is this pharmacy linked with any organization that provides family planning methods and materials at a discounted rate or for free (such as PPFN or SFH)?	Yes………………………………..1<br>No…………………………………2<br>Don't know………………………8	 →Q40<br>→Q40
Q39a.	What is the name of the organization?	Q39b. What year did this facility begin to associate with each organization named?	
	1.	Year ……………... . . . [ ][ ][ ][ ]<br>Don't know 9998	
	2.	Year ……………... . . . [ ][ ][ ][ ]<br>Don't know 9998	
	3.	Year ……………... . . . [ ][ ][ ][ ]<br>Don't know 9998	
	4.	Year ……………... . . . [ ][ ][ ][ ]<br>Don't know 9998	
Q40.	Organizations like SFH and PPFN sometimes distribute products at a lower price to pharmacies to sell. These are called socially marketed products.<br>Do you have socially marketed contraceptive products in stock?	Yes . . . . 1<br>No . . . . 2<br>Don't know 8	 →Q42<br>→Q42
Q41.	What are all the socially marketed family planning products that you have in stock? **LIST SPECIFIC FAMILY PLANNING BRAND NAMES.**<br><br>**(CODE WILL BE PROVIDED AT THE OFFICE)**	________________________[__|__|__]<br>________________________[__|__|__]<br>________________________[__|__|__]	
**STORAGE & STOCK:** Now I would like to see the place where contraceptive methods are stored. We are just trying to get an idea of how we can help outlets improve their stocking and storing methods. Remember that my findings will be just used for research purposes and will be kept strictly confidential.			
Q42.	OBSERVE WHETHER ALL THE CONTRACEPTIVE METHODS ARE PROTECTED FROM WATER OR DAMPNESS	YES . . . . 1<br>NO . . . . 2<br>CANNOT OBSERVE STORAGE AREA……………...………………..3	 <br><br>→ **Q48**
Q43.	OBSERVE WHETHER ALL THE CONTRACEPTIVE METHODS ARE OFF THE FLOOR	YES . . . . 1<br>NO 2	
Q44.	OBSERVE WHETHER THE CEILING ABOVE THE CONTRACEPTIVE METHODS IS INTACT AND NOT LEAKING	YES . . . . 1<br>NO 2	
Q45.	OBSERVE WHETHER ALL THE CONTRACEPTIVE METHODS ARE PROTECTED FROM THE SUN.	YES . . . . 1<br>NO 2	
Q46.	OBSERVE WHETHER THE ROOM IS CLEAN OF EVIDENCE OF RODENTS (BATS, RATS) OR PESTS (ROACHES, ETC).	YES . . . . 1<br>NO 2	
Q47.	OBSERVE WHETHER THE INJECTABLES ARE STORED UPRIGHT.<br><br>CHECK IF THE ARROW ON THE INJECTABLE IS FACING UP	YES . . . . 1<br>NO . . . . 2<br>NOT APPLICABLE/DON'T PROVIDE INJECTABLES………..7	
Q48.	Does the shop separate damaged and/or expired family planning methods from the usable products, and remove them from the inventory?<br><br>IF YES, ASK TO SEE EVIDENCE OF EACH OF THE INDICATED PRACTICES AND ALL THAT WERE OBSERVED. ALSO ASK FOR THE TALLY CARD TO CHECK FOR RECORDED BALANCE.	YES, DAMAGED/EXPIRED ITEM REMOVED FROM INVENTORY . . . ……………………. …... . . . . 1<br><br>REMOVED FROM SHELVES AND NO EXPIRED ITEMS PRESENT………………………………..…. ... . 2<br><br>EXPIRED ITEMS OBSERVED . . . . . . ……. .. 3<br><br>REPORTED YES BUT CANNOT OBSERVE….4<br><br>NO 5	

Q49.	OBSERVE WHETHER THERE IS A FUNCTIONAL REFRIGERATOR IN THE SHOP FOR STORING MEDICINES	YES, OBSERVED REFRIGERATOR AND FUNCTIONAL……………………..…………………......1<br><br>YES, OBSERVED REFRIGERATOR BUT NOT FUNCTIONAL OR NOT USED FOR STORING MEDICINES…... ………………………………..………..2<br><br>YES, BUT REFRIGERATOR NOT OBSERVED……..3<br><br>NO REFRIGERATOR PRESENT………………..…….5	

Q50.	RECORD THE TIME	Hour ………… ☐☐ Minutes …..☐☐
Thank you very much for taking the time to answer my questions. Once again, any information you have given will be kept confidential. Have a good day!		
COMMENTS:		
